# Pharmacogenetics of tenofovir and emtricitabine penetration into cerebrospinal fluid

**DOI:** 10.4102/sajhivmed.v22i1.1206

**Published:** 2021-04-28

**Authors:** Eric H. Decloedt, Phumla Z. Sinxadi, Lubbe Wiesner, John A. Joska, David W. Haas, Gary Maartens

**Affiliations:** 1 Division of Clinical Pharmacology, Department of Medicine, Faculty of Medicine and Health Sciences, Stellenbosch University, Cape Town, South Africa; 2 Division of Clinical Pharmacology, Department of Medicine, Faculty of Health Sciences, University of Cape Town, Cape Town, South Africa; 3 Department of Psychiatry and Mental Health, Health Sciences, Groote Schuur Hospital, University of Cape Town, Cape Town, South Africa; 4 Department of Medicine, Vanderbilt University Medical Center, Vanderbilt University, Nashville, Tennessee, United States; 5 Department of Internal Medicine, Meharry Medical College, Nashville, Tennessee, United States

**Keywords:** pharmacokinetics, pharmacogenetics, tenofovir, emtricitabine, cerebrospinal fluid

## Abstract

**Background:**

Blood-cerebrospinal fluid (CSF) barrier transporters affect the influx and efflux of drugs. The antiretrovirals tenofovir and emtricitabine may be substrates of blood-brain barrier (BBB) and blood-CSF barrier transporters, but data are limited regarding the pharmacogenetics and pharmacokinetics of their central nervous system (CNS) penetration.

**Objectives:**

We investigated genetic polymorphisms associated with CSF disposition of tenofovir and emtricitabine.

**Method:**

We collected paired plasma and CSF samples from 47 HIV-positive black South African adults who were virologically suppressed on efavirenz, tenofovir and emtricitabine. We considered 1846 single-nucleotide polymorphisms from seven relevant transporter genes (*ABCC5, ABCG2, ABCB1, SLCO2B1, SCLO1A2, SLCO1B1* and *ABCC4*) and 782 met a linkage disequilibrium (LD)-pruning threshold.

**Results:**

The geometric mean (95% confidence interval [CI]) values for tenofovir and emtricitabine CSF-to-plasma concentration ratios were 0.023 (0.021–0.026) and 0.528 (0.460–0.605), respectively. In linear regression models, the lowest *p*-value for association with the tenofovir CSF-to-plasma ratio was *ABCB1* rs1989830 (*p* = 1.2 × 10^−3^) and for emtricitabine, it was *ABCC5* rs11921035 (*p* = 1.4 × 10^−3^). None withstood correction for multiple testing.

**Conclusion:**

No genetic polymorphisms were associated with plasma, CSF concentrations or CSF-to-plasma ratios for either tenofovir or emtricitabine.

## Introduction

Tenofovir and emtricitabine are part of the current first-line antiretroviral therapy (ART) regimens for HIV-positive adults in resource-limited settings and both are widely used in high-income countries.^1^ Infection of the central nervous system (CNS) by HIV-1 occurs early in infection and its clearance is reliant on adequate CNS antiretroviral concentrations.^2^ However, there are limited data regarding determinants of cerebrospinal fluid (CSF) penetration by tenofovir and emtricitabine. Data from small cohorts indicate that CSF concentrations of tenofovir and emtricitabine are 5% and 50% of plasma concentrations, respectively.^3,4,5^

However, higher CSF tenofovir concentrations and lower emtricitabine concentrations have been reported, which may be explained by polymorphisms in drug transporters or altered blood–brain barrier (BBB) permeability.^4,5^ Transporters in the BBB and blood–CSF barrier (BCB) affect the influx and efflux of drugs, including tenofovir and emtricitabine.^3,6,7^ Multidrug resistance protein-5 (MRP-5, encoded by *ABCC5*) is ubiquitous and mediates the efflux of nucleoside reverse transcriptase inhibitors.^8^ Lower CSF emtricitabine exposure in females compared to males is hypothesised to reflect differential expression of MRP transporters at the BBB and BCB.^3^ *In vitro*, tenofovir is a substrate of the breast cancer resistance protein (BCRP, encoded by *ABCG2*), MRP-4 (encoded by *ABCC4*) and P-glycoprotein (encoded by *ABCB1*).^9,10,11^ A polymorphism in *ABCG2* rs2231142 has been associated with 1.5-fold increased plasma tenofovir exposure and Thai patients carrying *ABCC4* 3463 AG or GG (rs1751034) had an 11% greater tenofovir clearance compared with AA.^12,13^

Loss-of-function *ABCC4* polymorphisms have been associated with reduced clearance of tenofovir.^11,14^ In genome-wide analyses, *SLC17A1* rs12662869 was associated with an increase in tenofovir clearance.^15^ It is possible that genetic polymorphisms that affect transporter function will affect tenofovir or emtricitabine CSF penetration. The pharmacogenetics of CSF penetration of tenofovir and emtricitabine have not been described.

Africans are the most genetically diverse population worldwide.^16^ South Africa has the world’s largest ART programme, with most patients currently receiving efavirenz-based regimens that include the nucleos(t)ides tenofovir and emtricitabine.^17^ We previously reported on the pharmacogenetics of CSF penetration of efavirenz in black South Africans.^18^ Here, we characterise the associations between transporter gene polymorphisms and CSF penetration of tenofovir and emtricitabine in the same cohort.

## Patients and methods

### Participants

Adults (≥ 18 and ≤ 70 years of age) from a randomised control trial (PACTR201310000635418) that investigated lithium for HIV-associated neurocognitive impairment were invited to participate in the present study.^19^ We also invited participants who were screened for that trial but were excluded based on cognitive impairment criteria. All participants provided written informed consent. This study was approved by the University of Cape Town Human Research Ethics Committee (HREC 071/2013).

### Pharmacokinetic sampling

We collected paired plasma and CSF samples for tenofovir and emtricitabine assays. Participants recorded the dosing time the night before and were admitted in the morning for pharmacokinetic sampling. Whole blood was collected within 45 min of CSF sampling and centrifuged within 1 h of collection. Plasma and CSF aliquots were stored at −80 °C until analysis.

### Tenofovir and emtricitabine measurement

The analytical laboratory in the Division of Clinical Pharmacology at the University of Cape Town quantified total tenofovir and emtricitabine in plasma and CSF using validated liquid chromatography tandem mass spectrometry assays.

The lower limits of quantification (LLQs) for plasma tenofovir and emtricitabine were 10.0 ng/mL and 37.5 ng/mL, respectively. For CSF, the LLQs for total tenofovir and emtricitabine were 0.5 ng/mL. Concentrations below the limits of quantification were treated as missing data.

### Characterisation of genetic polymorphisms

We extracted DNA from the buffy coat using the QIAsymphony kit. Genotyping was performed using the Infinium® Expanded Multi-Ethnic Genotyping Array (MEGA^EX^; Illumina, San Diego, CA, USA).

Polymorphisms that were not genotyped were imputed. Polymorphisms were extracted from seven genes ±50 kB: *ABCB1* (301 polymorphisms), *ABCC4* (630 polymorphisms), *ABCC5* (225 polymorphisms), *ABCG2* (164 polymorphisms), *SLCO1A2* (406 polymorphisms) and *SLCO2B1* (118 polymorphisms). Polymorphisms were excluded for genotyping efficiency less than 99%, minor allele frequency less than 5% and Hardy-Weinberg equilibrium *p*-values less than 0.00001. We also genotyped *SLCO1B1* 521T→C (rs4149056) and *SLCO1B1* (rs4149032) using the MassARRAY iPLEX® Gold system (Sequenom, Inc., San Diego, CA, USA).

All genotyping was performed at Vanderbilt Technologies for Advanced Genomics (VANTAGE), by laboratory personnel with no knowledge of clinical data. All samples were genotyped in duplicate. The final dataset included 1846 polymorphisms from 47 participants.

### Pharmacokinetic statistical analysis

Pharmacokinetic data were not normally distributed so were expressed as median and interquartile ranges (IQRs) and geometric means (95% confidence interval [CI]). Pearson’s *r* correlation was used to assess the correlations between plasma and CSF concentrations. We performed statistical analysis using STATA version 15.0 (StataCorp, College Station, TX, USA). Graphs were created using GraphPad Prism version 7.03 for Windows (GraphPad Software, La Jolla, CA, USA).

### Genetic associations

Associations with pharmacokinetic parameters were assessed by univariable analysis. Pharmacokinetic data were log_10_ transformed for association analyses. We used ratios of total concentrations without correcting for protein binding. Cerebrospinal fluid-to-plasma concentration ratios were calculated using raw concentrations and then log_10_ transformed. We performed genetic association analyses using PLINK version 1.9.^20^

For primary analyses, we conducted linkage disequilibrium (LD) pruned with an R^2^ threshold of 0.95 within a 50-kB window at 5-kB increments.

The final analysis included 782 polymorphisms that met the LD-pruning threshold. We used Bonferroni correction to adjust for multiple testing (*p* = 0.05 divided by 782 polymorphisms). We generated an LD plot using Haploview (https://www.broadinstitute.org/haploview/haploview). We previously reported LD plots for these polymorphisms.^18^

### Ethical considerations

All participants provided written informed consent. This study was approved by the University of Cape Town Human Research Ethics Committee (HREC 071/2013).

## Results

We studied 47 participants who self-identified as black South Africans (isiXhosa speaking), of whom 41 were female. All were virologically suppressed and were receiving efavirenz, tenofovir and emtricitabine (*n* = 43) or efavirenz, tenofovir and lamivudine (*n* = 4). The median (IQR) values of the baseline characteristics were age 36 (IQR = 32–43) years, a CD_4_ T-cell count of 470 (IQR = 384–586) cells/mm^3^, a time on ART of 38 (IQR = 18–54) months and a body mass index (BMI) of 25.6 (IQR = 22.7–29.3) kg/m^2^. The concentrations of tenofovir (plasma and CSF) and emtricitabine (plasma and CSF) are presented in Table 1. The plasma and CSF concentrations of tenofovir and emtricitabine were each correlated (*p* < 0.0001, R^2^ = 0.53 and *p* < 0.0001, R^2^ = 0.45; respectively) (Appendix Figure 1a and 1b). There was no statistically significant association of CSF-to-plasma ratios versus time after dosing (Appendix Figure 2).

**TABLE 1:** Concentrations of tenofovir and emtricitabine in plasma and cerebrospinal fluid.

Nucleos(t)ides	Data for											
	Plasma				CSF				CSF-to-plasma ratio			
	*Value*	IQR	%	CI	*Value*	IQR	%	CI	*Value*	IQR	%	CI
**Tenofovir (*n* = 47)**												
BLQ samples, *n* (%)	3	-	6.4	-	4	-	8.5	-	4	-	8.5	-
Median concentration, ng/mL (IQR)	63.5	50.8–81.2	-	-	1.4	1.1–2.1	-	-	0.023	0.018–0.030	-	-
Geometric mean, ng/mL (95% CI)	62.57	-	-	53.91–72.63	1.49	-	-	1.28–1.73	0.023	-	-	0.021–0.026
Range, ng/mL	23–246	-	-	-	0.51–5.3	-	-	-	0.012–0.047	-	-	-
**Emtricitabine (*n* = 43)**												
BLQ samples, *n* (%)	4	-	17	-	4	-	9	-	4	-	17	-
Median concentration, ng/mL (IQR)	139	109–166	-	-	63.5	47.4–102	-	-	0.534	0.392–0.675	-	-
Geometric mean, ng/mL (95% CI)	133.7	-	-	112.1–159.4	56.0	-	-	41.7–75.0	0.528	-	-	0.460–0.605
Range, ng/mL	39.3–560	-	-	-	1.5–167	-	-	-	0.207–2.007	-	-	-

CSF, cerebrospinal fluid; IQR, interquartile range; 95% CI, 95% confidence interval; BLQ, below limits of assay quantification.

### Genetic polymorphisms

Amongst the 47 participants, 1846 polymorphisms were successfully genotyped. Only *SLCO1B1* rs4149056 was monomorphic (i.e. no minor alleles). The remaining 1845 polymorphisms were in Hardy–Weinberg equilibrium based on a Bonferroni-adjusted *p*-value threshold of 6.4 × 10^−5^; 56 had unadjusted Bonferroni *p*-values of < 0.05. Minor allele frequencies for all polymorphisms are provided in Appendix Table 1.

### Genetic associations with tenofovir and emtricitabine cerebrospinal fluid penetration

In univariable linear regression analyses (Table 2), the tenofovir CSF-to-plasma ratio was best predicted by a model that included *ABCB1* rs1989830 (β = −0.12; 95% CI = −0.19 – –0.05; *p* = 1.2 × 10^−3^). The emtricitabine CSF-to-plasma ratio was best predicted by a model that included *ABCC5* rs11921035 (β = −0.32; 95% CI = −0.50 – –0.14; *p* = 1.4 × 10^−3^), as shown in Table 3. No association achieved significance after correcting for multiple testing. Univariable linear regression analyses and polymorphisms with *p*-values below 0.01 for tenofovir and emtricitabine CSF-to-plasma ratios are shown in Tables 2 and 3, respectively. For absolute plasma and CSF tenofovir concentrations, 10 polymorphisms in *ABCG2, ABCC5, SLCO1A2* and *ABCC4* for plasma and six in *ABCB1, ABCG2, ABCC5, SLCO1A2* and *ABCC4* for CSF had *p*-values less than 0.01 (data not shown). For absolute plasma and CSF emtricitabine concentrations, six polymorphisms in *ABCC5, SLCO1A2, ABCC4* and *SLCO2B1* for plasma and 12 in *ABCB1, ABCG2, ABCC5, SLCO1A2* and *ABCC4* had *p*-values less than 0.01 (data not shown). No associations with *SLCO1B1* rs4149032 were found.

**TABLE 2:** Genetic associations with detectable log10-transformed cerebrospinal fluid-to-plasma tenofovir concentrations in 43 black South African adults.

Chromosome	Gene	Polymorphism	Minor allele frequency	Beta coefficient	95% CI	p*
7	*ABCB1*	rs1989830	0.30	−0.12	−0.19–−0.05	1.2 × 10^−3^
7	*ABCB1*	rs78551545	0.06	−0.28	−0.43–−0.12	1.3 × 10^−3^
12	*SLCO1A2*	rs11535999	0.33	0.13	0.05–0.21	2.5 × 10^−3^
4	*ABCG2*	rs111917717	0.05	−0.28	−0.46–−0.11	3.0 × 10^−3^
4	*ABCG2*	rs76462878	0.08	−0.22	−0.36–−0.08	4.0 × 10^−3^
7	*ABCB1*	rs35572298	0.08	−0.18	−0.30–−0.06	5.0 × 10^−3^
12	*SLCO1A2*	rs4149008	0.38	0.11	0.03–0.18	6.9 × 10^−3^
12	*SLCO1A2*	rs4149009	0.32	0.11	0.03–0.19	7.8 × 10^−3^
12	*SLCO1A2*	rs10841786	0.33	0.11	0.03–0.19	7.8 × 10^−3^
12	*SLCO1A2*	rs57472326	0.33	0.11	0.03–0.19	7.8 × 10^−3^
12	*SLCO1A2*	rs7968842	0.34	0.11	0.03–0.19	9.2 × 10^−3^

95% CI, 95% confidence interval.

* , Bonferroni-corrected *p*-value cut-off = 6.4 × 10^−5^.

**TABLE 3:** Genetic associations with detectable log_10_-transformed cerebrospinal fluid -to plasma emtricitabine concentrations in 39 South African adults.

Chromosome	Gene	Polymorphism	Minor allele frequency	Beta coefficient	95% CI	p*
3	*ABCC5*	rs11921035	0.08	−0.32	−0.50–−0.14	1.4 × 10^−3^
12	*SLCO1A2*	rs4762700	0.48	−0.16	−0.25–−0.06	2.0 × 10^−3^
3	*ABCC5*	rs11928606	0.09	−0.27	−0.44–−0.10	4.4 × 10^−3^
13	*ABBC4*	rs7322318	0.34	0.13	0.05–0.22	5.3 × 10^−3^
13	*ABCC4*	rs9590228	0.38	0.13	0.04–0.22	6.5 × 10^−3^
13	*ABCC4*	rs4148428	0.21	−0.18	−0.30–−0.06	7.4 × 10^−3^
3	*ABCC5*	rs116312201	0.13	0.19	0.06–0.33	9.0 × 10^−3^
4	*ABCG2*	rs1448784	0.05	−0.32	−0.54–−0.09	9.3 × 10^−3^

95% CI, 95% confidence interval.

* , Bonferroni-corrected *p*-value cut-off = 6.4 × 10^−5^.

## Discussion

We characterised the associations between 782 genetic polymorphisms and CSF disposition of tenofovir and emtricitabine in black South African adults. The lowest *p*-value for tenofovir CSF-to-plasma ratio was *ABCB1* rs1989830 (*p* = 1.2 × 10^−3^), and for emtricitabine was *ABCC5* rs11921035 (*p* = 1.4 × 10^−3^). None were significant after correcting for multiple testing. In addition, we found no significant associations with absolute CSF or plasma concentration after correcting for multiple testing.

Associations with tenofovir pharmacokinetics and genetic polymorphisms were found in other populations. An increase in tenofovir plasma concentrations were independently associated with *ABCC4* 4131T→G (genotype TG or GG) in 150 Thai HIV-infected adults.^14^ *ABCB1* rs3213619 was associated with increased tenofovir bioavailability in a predominantly African-American patient population (*n* = 45) and thought to be a result of decreased P-glycoprotein function.^21^ A genome-wide and candidate gene association analyses with tenofovir pharmacokinetics showed that *SLC17A1* rs12662869 was associated with an increase in tenofovir clearance (*p* = 7.1 × 10^−9^) but failed to show significant associations in candidate genes (including *ABCC4, ABCC10, ABCB1, ABCC2, SLC22A11, AK2* and *AK3*) after correction for multiple comparisons.^15^

Our study has limitations. With our sample size, we were underpowered to detect associations with small effect sizes. We could only detect associations with relatively frequent polymorphisms and with large effect sizes. Therefore, these data should be regarded as exploratory. Polymorphisms not genotyped in our study may be associated with tenofovir or emtricitabine disposition into CSF. Whilst we did not adjust for creatinine clearance, this should not be a confounder that affects drug disposition into CSF. We included 33 (70%) participants with mild to moderate neurocognitive impairment, as previously reported.^18^ We may therefore have introduced a selection bias.

## Conclusion

In conclusion, we found no significant associations between any of the 782 polymorphisms and plasma concentrations, CSF concentrations or CSF-to-plasma ratios for either tenofovir or emtricitabine in univariate linear regression models after correcting for multiple testing.

## Figures and Tables

**FIGURE 1-A1:**
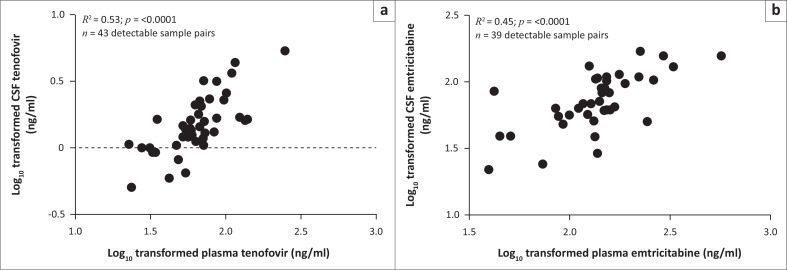
Pearson correlation plots for log_10_-transformed plasma and cerebrospinal fluid (CSF) concentrations emtricitabine and tenofovir. *Panel A*: Relationship between CSF and plasma tenofovir concentrations. *Panel B*: Relationship between CSF and plasma emtricitabine concentrations. All concentrations are log_10_ transformed.

**FIGURE 2-A1:**
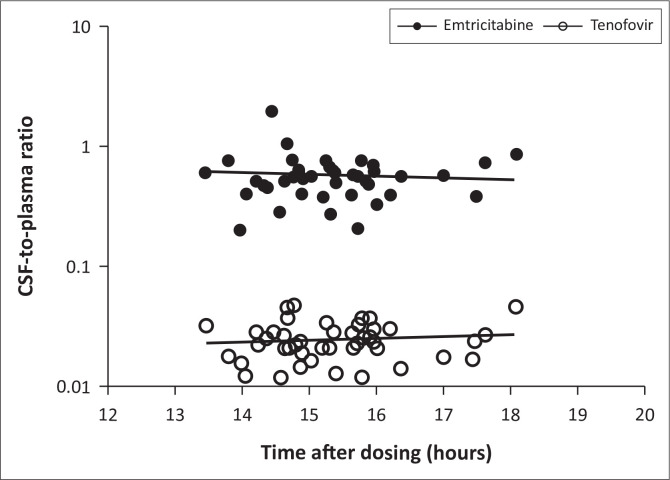
Cerebrospinal fluid (CSF)-to-plasma concentration ratios of detectable pairs of plasma and CSF samples versus time after dosing. The lines are linear regression lines and were not statistically significant.

## Appendix

**TABLE 1-A1:** Minor allele frequencies for 1846 polymorphisms in 43 black South Africans.

Chromosome	Polymorphism	Minor allele	Major allele	Minor allele frequency
3	rs73887541	C	G	0.1744
3	rs142034818	A	G	0.1744
3	rs11928605	T	C	0.0697
3	rs11928606	T	C	0.0930
3	rs6443917	T	C	0.3721
3	rs11921035	G	A	0.0814
3	rs11914385	T	C	0.0814
3	rs11914437	A	G	0.0930
3	rs6776999	G	T	0.3605
3	rs115851049	A	T	0.1744
3	rs7635679	T	C	0.4535
3	rs7648408	A	T	0.3605
3	rs7648487	A	G	0.3605
3	rs113227666	T	C	0.0930
3	rs12696511	A	G	0.1279
3	rs111485412	T	C	0.0814
3	rs5855004	AT	A	0.3721
3	rs9290776	C	A	0.3605
3	rs73044605	A	G	0.0814
3	rs188783928	C	T	0.0814
3	rs149185640	A	AAGAG	0.0814
3	rs75086702	T	C	0.1744
3	rs11926985	A	G	0.2326
3	rs56772648	G	C	0.1744
3	rs56333370	G	A	0.1744
3	rs35494670	G	T	0.4419
3	rs35717944	A	C	0.2558
3	rs6792309	T	C	0.4767
3	rs75822913	A	G	0.0930
3	rs1879256	G	A	0.4884
3	rs1879255	A	C	0.314
3	rs1879254	T	C	0.3023
3	rs56874920	T	TA	0.2093
3	rs73887563	A	G	0.1744
3	rs73887565	A	G	0.186
3	rs2139558	G	A	0.3953
3	rs35369718	G	A	0.3953
3	rs17683012	T	C	0.3953
3	rs1012987	G	T	0.3953
3	rs1012988	A	G	0.3953
3	rs1012989	T	G	0.3953
3	rs872013	A	G	0.2442
3	rs73044669	C	T	0.2326
3	rs7434228	T	C	0.2558
3	rs2176825	A	G	0.2558
3	rs1533682	T	C	0.2326
3	rs1533683	C	T	0.3488
3	rs73044685	T	G	0.0581
3	rs1000002	T	C	0.1744
3	rs61698626	T	C	0.2907
3	rs1533684	A	G	0.2326
3	rs2313217	A	G	0.2326
3	rs6768149	C	T	0.1744
3	rs2872249	T	A	0.2442
3	rs79625907	T	C	0.1047
3	rs73044690	C	A	0.0930
3	rs3805114	G	T	0.1047
3	rs562	T	C	0.3372
3	rs3749445	T	C	0.1744
3	rs73884807	A	G	0.2442
3	rs60053269	G	GA	0.2326
3	rs112357545	T	C	0.0581
3	rs7646621	G	T	0.4767
3	rs77093210	A	G	0.1047
3	rs13317532	G	C	0.2442
3	rs113699660	C	G	0.0581
3	rs113422085	T	C	0.0697
3	rs111666830	C	T	0.0581
3	rs113311136	C	T	0.0581
3	rs2139562	G	A	0.2907
3	rs4148593	G	A	0.5
3	rs6766986	C	G	0.2442
3	rs112840402	T	C	0.0581
3	rs116312201	A	G	0.1279
3	rs147028569	T	C	0.0581
3	rs113115970	T	C	0.0581
3	rs11714326	T	C	0.2209
3	rs13064234	A	G	0.2209
3	rs113010279	C	T	0.0581
3	rs6790814	G	C	0.2093
3	rs111868993	C	T	0.0581
3	rs111248225	T	C	0.0581
3	rs6443922	G	A	0.2209
3	rs6804242	A	T	0.2326
3	rs59518404	A	G	0.2209
3	rs115503048	G	T	0.0581
3	rs3749442	A	G	0.2209
3	rs73884809	G	T	0.2442
3	rs73046532	C	G	0.1047
3	rs10575785	G	GAAC	0.2093
3	rs73046540	C	T	0.1279
3	rs6443923	A	C	0.3488
3	rs6805913	T	G	0.2326
3	rs9812777	T	C	0.2093
3	rs4148589	C	T	0.2209
3	rs2292998	T	C	0.2326
3	rs9818518	T	C	0.2209
3	rs9838667	G	T	0.3488
3	rs3840256	GAT	G	0.2209
3	rs6799583	C	G	0.2209
3	rs3817404	A	C	0.1628
3	rs189855476	C	T	0.0581
3	rs2139564	A	G	0.2209
3	rs111952578	C	G	0.0581
3	rs59309690	A	G	0.0930
3	rs115663763	A	T	0.0581
3	rs116483375	T	C	0.0581
3	rs1401999	C	G	0.2209
3	rs6443924	A	G	0.2442
3	rs149330260	A	G	0.0581
3	rs6766401	T	C	0.2209
3	rs6443925	C	G	0.4535
3	rs6766526	T	C	0.2209
3	rs6767013	G	A	0.2209
3	rs187791141	C	T	0.0581
3	rs6767306	A	G	0.2209
3	rs1879257	A	G	0.2209
3	rs3792583	A	G	0.2209
3	rs4148579	T	C	0.2209
3	rs939336	A	G	0.2442
3	rs6800217	T	C	0.2442
3	rs35240483	A	C	0.2209
3	rs112737137	T	C	0.2209
3	rs7636305	A	G	0.2209
3	rs9861983	T	C	0.2209
3	rs869417	T	C	0.2209
3	rs28365012	A	G	0.0581
3	rs3828469	G	A	0.2209
3	rs3805108	T	C	0.3372
3	rs73179688	T	C	0.4419
3	rs144452121	C	T	0.0581
3	rs113468175	C	G	0.0581
3	rs144412198	A	G	0.0581
3	rs3792581	A	C	0.2209
3	rs114398776	G	A	0.0581
3	rs4148578	A	C	0.2209
3	rs4148577	C	T	0.2209
3	rs1132776	A	G	0.3372
3	rs6775518	A	G	0.2209
3	rs6791345	T	C	0.3372
3	rs6802849	A	C	0.2209
3	rs55695073	T	C	0.2093
3	rs7636910	C	T	0.186
3	rs2293001	T	C	0.4419
3	rs2313212	A	G	0.2442
3	rs939337	G	C	0.2209
3	rs75617395	T	C	0.0930
3	rs3749440	G	A	0.4419
3	rs4148575	A	G	0.3372
3	rs6795595	T	C	0.2558
3	rs57777233	T	C	0.2558
3	rs3749438	A	G	0.186
3	rs112403322	G	C	0.0581
3	rs6443926	A	C	0.3372
3	rs73884816	G	A	0.2558
3	rs7635948	C	G	0.3372
3	rs10937157	A	G	0.2791
3	rs12634398	G	A	0.4419
3	rs10937158	T	C	0.2791
3	rs113691647	A	ACAAAAGTGCATG	0.2096
3	rs11404217	GA	G	0.3953
3	rs75393197	G	T	0.0581
3	rs28680881	G	T	0.1628
3	rs7620350	A	T	0.2791
3	rs7620781	A	G	0.2907
3	rs138640574	A	C	0.2558
3	rs35740940	G	C	0.0697
3	rs77059190	T	C	0.2558
3	rs75448974	A	G	0.2442
3	rs73884819	T	G	0.2442
3	rs9290779	A	C	0.1628
3	rs145536424	T	A	0.0581
3	rs55831983	C	T	0.1047
3	rs1879259	A	G	0.2209
3	rs4148557	A	G	0.3256
3	rs7610724	G	T	0.0697
3	rs7613237	C	T	0.407
3	rs939335	A	G	0.2791
3	rs11917442	T	C	0.0581
3	rs201188880	CT	C	0.2442
3	rs11711219	C	A	0.2558
3	rs10937159	A	C	0.2558
3	rs7622660	C	T	0.0930
3	rs36092077	A	G	0.1628
3	rs141952918	G	A	0.0581
3	rs6792482	T	C	0.2442
3	rs6779545	C	A	0.2442
3	rs6443930	G	C	0.2442
3	rs111689452	A	G	0.0348
3	rs1000952	G	A	0.1163
3	rs6765152	A	C	0.0814
3	rs56006127	A	G	0.0465
3	rs7621975	G	A	0.4186
3	rs56060490	C	A	0.0465
3	rs55741914	G	A	0.0814
3	rs57484150	T	C	0.0814
3	rs10937161	T	C	0.1977
3	rs58413046	C	T	0.0697
3	rs60791004	A	G	0.1628
3	rs7430671	C	G	0.3605
3	rs73183426	G	C	0.1977
3	rs74763842	T	G	0.4186
3	rs116348145	T	C	0.0814
3	rs56889675	T	G	0.2558
3	rs10470524	T	G	0.2209
3	rs7641834	T	C	0.1977
3	rs115021567	G	A	0.1977
3	rs60179621	A	G	0.1977
3	rs6762938	T	C	0.314
3	rs6794223	G	A	0.1395
3	rs7616916	A	C	0.0814
3	rs75574047	C	A	0.0814
3	rs6807271	A	G	0.314
3	rs6807670	A	G	0.3953
3	rs13094369	A	G	0.3953
3	rs7610344	T	G	0.2791
3	rs7427051	A	G	0.2442
3	rs28476779	G	A	0.2791
3	rs9851097	A	G	0.2791
3	rs11924955	T	C	0.2558
3	rs60964651	AT	A	0.2791
3	rs79813226	A	C	0.2442
3	rs6779862	A	C	0.2442
3	rs6443933	G	A	0.3605
3	rs13320474	A	G	0.2791
4	rs2725215	T	C	0.1395
4	rs2728108	A	C	0.0814
4	rs111917717	T	A	0.0465
4	rs17013754	G	A	0.1163
4	rs2728107	T	C	0.0465
4	rs17013761	T	C	0.1163
4	rs6843032	T	G	0.4767
4	rs113152807	T	C	0.0465
4	rs7672253	A	G	0.0581
4	rs116685328	T	C	0.0465
4	rs4336187	A	G	0.2791
4	rs2728121	T	C	0.2093
4	rs10965	A	G	0.1395
4	rs74712548	A	C	0.1047
4	rs201502076_t3	T	C	0.4767
4	rs45542445	A	T	0.0581
4	rs115770495	T	C	0.0581
4	rs1448784	G	A	0.0465
4	rs4148159	T	A	0.1744
4	rs2231164	T	C	0.2907
4	rs2725267	A	G	0.3023
4	rs2231162	A	G	0.314
4	rs2231159	C	A	0.2093
4	rs2231158	C	T	0.2093
4	rs45621036	C	T	0.1628
4	rs45556834	G	A	0.2093
4	rs45469894	C	T	0.1628
4	rs1383586	G	A	0.3023
4	rs1383584	A	G	0.3023
4	rs45592333	C	T	0.1628
4	rs10433946	C	T	0.0581
4	rs2231155	T	C	0.1744
4	rs45566934	T	C	0.1628
4	rs141097556	C	G	0.1628
4	rs2622614	T	C	0.4651
4	rs2622613	A	G	0.314
4	rs45443398	T	C	0.1163
4	rs2231153	T	C	0.3023
4	rs141518597	T	G	0.0814
4	rs201742138	AG	A	0.1163
4	rs28665233	A	G	0.1395
4	rs2725264	T	C	0.1395
4	rs2725263	C	A	0.1512
4	rs2622628	A	C	0.3256
4	rs12505410	G	T	0.0814
4	rs2622621	G	C	0.0581
4	rs13120400	C	T	0.0697
4	rs201460174	G	GTCTCTCTCTCTCTCTGTC	0.0814
4	rs199994188	C	CTCTCTCTCTCTCTG	0.0814
4	rs57892861	C	T	0.314
4	rs6532048	G	A	0.0814
4	rs2231147	C	T	0.1395
4	rs1871744	C	T	0.0814
4	rs2622618	A	G	0.0465
4	rs2231144	C	T	0.3372
4	rs113752350	C	T	0.3372
4	rs185151667	T	C	0.3372
4	rs2725259	T	C	0.0465
4	rs6832558	T	C	0.0465
4	rs2725258	T	C	0.0465
4	rs2725256	G	A	0.3372
4	rs45488400	C	T	0.3372
4	rs17013859	T	C	0.3372
4	rs200576598	AG	A	0.0581
4	rs2725255	A	G	0.0581
4	rs2622619	G	C	0.0465
4	rs17013870	C	T	0.3372
4	rs72875335	A	G	0.3372
4	rs113737399	A	G	0.314
4	rs2622631	G	A	0.0581
4	rs2622632	G	A	0.0465
4	rs12641369	A	G	0.4651
4	rs2725253	C	T	0.0581
4	rs2622617	G	A	0.0581
4	rs1564481	T	C	0.1628
4	rs2725252	A	C	0.2907
4	rs13130891	A	G	0.5
4	rs4148149	T	G	0.4651
4	rs13137622	G	T	0.5
4	rs2046134	A	G	0.4186
4	rs2725250	G	A	0.2326
4	rs3114018	C	A	0.4651
4	rs3109823	T	C	0.5
4	rs17013881	T	C	0.1163
4	rs113094792	T	C	0.0465
4	rs2622627	A	C	0.4419
4	rs73844307	C	T	0.0465
4	rs2725249	C	A	0.4419
4	rs6857600	T	C	0.3605
4	rs115055824	G	A	0.0581
4	rs114085125	A	C	0.0581
4	rs114726329	T	C	0.0581
4	rs11934545	A	G	0.0581
4	rs2622626	A	C	0.4419
4	rs11287117	T	TG	0.4535
4	rs11413103	GT	G	0.4419
4	rs6532049	T	C	0.4767
4	rs2725247	A	G	0.2326
4	rs17731799	T	G	0.3605
4	rs2725246	A	G	0.2326
4	rs2725245	A	G	0.2326
4	rs2725244	C	T	0.4651
4	rs2622624	C	T	0.2326
4	rs2725242	A	T	0.4651
4	rs1466480	A	G	0.3488
4	rs6820121	C	T	0.0581
4	rs6843273	A	G	0.0581
4	rs6843542	T	C	0.0697
4	rs6844086	A	G	0.0581
4	rs2622604	T	C	0.1163
4	rs111766106	A	G	0.1279
4	rs45604438	T	G	0.0814
4	rs3114019	C	T	0.0697
4	rs3114020	C	T	0.1744
4	rs11732936	G	A	0.1628
4	rs10011796	T	C	0.314
4	rs28440048	T	C	0.1163
4	rs35228269	G	A	0.3488
4	rs7657441	C	T	0.1744
4	rs6837857	A	C	0.3488
4	rs35839768	G	A	0.3488
4	rs35537015	C	T	0.3488
4	rs34731996	C	T	0.3488
4	rs35229708	C	T	0.3488
4	rs35252139	T	C	0.3488
4	rs7699188	A	G	0.3488
4	rs7699279	T	C	0.3488
4	rs7657531	C	A	0.3488
4	rs7682521	G	T	0.3488
4	rs7658211	A	G	0.3488
4	rs7657757	G	A	0.3488
4	rs7682757	C	T	0.3488
4	rs7657928	C	A	0.3488
4	rs7658584	A	G	0.3488
4	rs112385917	CTTTA	C	0.3488
4	rs13128241	T	C	0.3488
4	rs13128083	G	A	0.3488
4	rs55987521	T	A	0.3721
4	rs60816576	T	G	0.3953
4	rs57454797	C	T	0.3953
4	rs1481014	A	C	0.3721
4	rs13135956	A	G	0.3721
4	rs76462878	T	A	0.0814
4	rs6821227	C	T	0.2442
4	rs6821239	A	G	0.2442
4	rs9784454	C	T	0.2442
4	rs6532053	A	G	0.2442
4	rs6833713	G	A	0.2442
4	rs6833950	G	A	0.2442
4	rs2127861	C	G	0.2442
4	rs2127863	T	C	0.2442
4	rs70959608	C	CA	0.2442
4	rs6854688	G	A	0.314
4	rs11097182	T	C	0.3837
4	rs113611770	C	A	0.0465
4	rs112710034	G	C	0.0465
4	rs6532055	C	T	0.1628
4	rs10856870	C	T	0.4651
4	rs150614746	G	A	0.0465
4	rs140027200	T	C	0.0465
4	rs75048878	T	G	0.1279
4	rs4693930	A	G	0.1047
4	rs139884402	G	C	0.0465
4	rs11723264	A	G	0.3953
7	rs67721532	G	A	0.1512
7	rs45505292	C	T	0.0930
7	rs112113287	G	GTCGTGTTT	0.407
7	rs60123540	C	T	0.407
7	rs17149637	A	G	0.407
7	rs17149640	C	A	0.407
7	rs17149641	C	T	0.407
7	rs45580239	A	G	0.2093
7	rs45546132	T	C	0.407
7	rs45447097	A	G	0.407
7	rs17149647	C	T	0.3837
7	rs45607141	G	A	0.407
7	rs17149652	T	G	0.407
7	rs4148817	G	C	0.407
7	rs4148815	T	A	0.407
7	rs66463970	GT	G	0.407
7	rs45593435	G	A	0.407
7	rs17149660	C	T	0.407
7	rs45502492	T	C	0.3605
7	rs45526438	A	G	0.3605
7	rs45605032	T	C	0.407
7	rs45590633	T	C	0.1512
7	rs45446703	A	G	0.0930
7	rs4148814	C	T	0.3605
7	rs2302385	C	T	0.3605
7	rs2302386	G	A	0.3605
7	rs2302387	A	G	0.407
7	rs7782643	A	G	0.1512
7	rs45564638	G	A	0.0465
7	rs2888611	G	C	0.407
7	rs4148808	C	T	0.2674
7	rs4148807	A	G	0.2674
7	rs4148805	A	G	0.2674
7	rs10556862	A	ATG	0.2209
7	rs73399961	G	A	0.0581
7	rs45555137	T	TTC	0.2209
7	rs45443201	C	T	0.0814
7	rs12673662	G	C	0.2209
7	rs66510518	G	C	0.2791
7	rs73705216	G	T	0.0814
7	rs6465116	A	G	0.2326
7	rs148890468	A	C	0.2326
7	rs11977881	C	T	0.2558
7	rs59934297	G	T	0.2791
7	rs58083477	C	T	0.2791
7	rs59616301	C	T	0.2791
7	rs7805184	A	G	0.2791
7	rs66869391	A	G	0.2791
7	rs17209662	A	G	0.2791
7	rs2178658	T	G	0.2093
7	rs17275514	G	A	0.2791
7	rs78551545	A	G	0.0581
7	rs998671	T	C	0.2674
7	rs12538707	C	T	0.2326
7	rs17209837	C	T	0.2791
7	rs150974072	A	AAT	0.2558
7	rs12672720	G	A	0.2326
7	rs60541816	A	G	0.2326
7	rs60916103	G	A	0.2326
7	rs6946119	C	T	0.0581
7	rs73198349	A	C	0.1163
7	rs7802555	C	A	0.2326
7	rs7802783	T	C	0.2326
7	rs3842	C	T	0.2442
7	rs28364278_t3	I	D	0.0465
7	rs28364277	T	C	0.1279
7	rs17064	A	T	0.2326
7	rs28401816	C	T	0.1628
7	rs28401814	A	G	0.1628
7	rs1882478	C	T	0.3837
7	rs2235048	G	A	0.0930
7	rs2235047	C	A	0.2093
7	rs1045642	A	G	0.0930
7	rs2229107	T	A	0.1047
7	rs10808071	G	A	0.2326
7	rs1002204	A	C	0.1395
7	rs17149699	T	C	0.3023
7	rs112292979	A	G	0.1047
7	rs4148751	C	T	0.1628
7	rs4148749	C	G	0.0581
7	rs111526989	T	G	0.0697
7	rs28401796	A	G	0.1744
7	rs112845881	C	T	0.0697
7	rs1922244	A	G	0.2674
7	rs113724043	T	C	0.0697
7	rs187721980	A	G	0.0697
7	rs3028303	TTC	T	0.2326
7	rs28401792	G	GATCCACTATGCCTA	0.1744
7	rs4148747	A	T	0.1977
7	rs4148746	G	GT	0.1977
7	rs4148745	T	G	0.1395
7	rs28401781	T	C	0.2442
7	rs147600670	A	AC	0.1744
7	rs2235067	T	C	0.1279
7	rs4148743	T	C	0.314
7	rs1882477	C	G	0.2326
7	rs2373589	T	C	0.3488
7	rs113822506	A	G	0.1047
7	rs4148740	G	A	0.186
7	rs147898841	C	T	0.0697
7	rs113521552	C	T	0.0697
7	rs10246696	A	G	0.186
7	rs11979702	A	T	0.1279
7	rs2141849	A	C	0.1628
7	rs112216837	G	C	0.0697
7	rs55912869	C	T	0.186
7	rs2373588	A	G	0.1628
7	rs10280101	C	A	0.186
7	rs35572298	A	AG	0.0814
7	rs150867018	G	A	0.0697
7	rs16885829	C	T	0.1395
7	rs67151359	G	GT	0.1628
7	rs10225473	G	A	0.1279
7	rs6971264	A	G	0.1395
7	rs35280822	A	G	0.0814
7	rs10240953	T	G	0.0697
7	rs7787082	G	A	0.2442
7	rs2373587	G	C	0.1628
7	rs113158842	G	T	0.0697
7	rs28681479	C	T	0.2093
7	rs2373585	T	C	0.1628
7	rs2032583	G	A	0.186
7	rs4148739	C	T	0.186
7	rs11983225	C	T	0.186
7	rs113106026	G	A	0.0697
7	rs10236274	G	A	0.2442
7	rs183410334	G	A	0.0697
7	rs11760837	C	T	0.186
7	rs139774375	A	G	0.0697
7	rs11972405	C	T	0.186
7	rs10274587	A	G	0.1512
7	rs28381959	A	G	0.1047
7	rs28381958	G	GA	0.3721
7	rs10248420	A	G	0.3721
7	rs149043325	A	G	0.0581
7	rs113764224	G	A	0.0814
7	rs2235040	T	C	0.1512
7	rs111992902	T	C	0.0697
7	rs79890058	A	G	0.0348
7	rs28381951	T	G	0.0348
7	rs12668877	T	C	0.2326
7	rs111538144	G	A	0.0697
7	rs3789246	T	C	0.2326
7	rs2235064	G	T	0.0697
7	rs7795817	T	C	0.2326
7	rs12154941	T	C	0.0814
7	rs28381940	G	A	0.1744
7	rs4148737	C	T	0.4651
7	rs4148736	A	G	0.4651
7	rs4728700	T	C	0.1977
7	rs28381933	G	A	0.0465
7	rs6961419	C	T	0.4651
7	rs6961882	C	T	0.4651
7	rs60503369	A	T	0.0465
7	rs6980101	T	C	0.2674
7	rs4148735	T	C	0.4651
7	rs1922242	T	A	0.4651
7	rs2235046	T	C	0.1628
7	rs2091766	T	C	0.4651
7	rs12704363	T	C	0.2093
7	rs28381909	A	G	0.2907
7	rs112489538	T	A	0.0697
7	rs10713802	A	AG	0.2907
7	rs115780656	T	C	0.1163
7	rs60412009	C	T	0.1279
7	rs113686230	A	G	0.1163
7	rs143422171	A	G	0.0465
7	rs2235013	T	C	0.4186
7	rs28381898	T	C	0.0465
7	rs2235035	A	G	0.1628
7	rs2235033	G	A	0.4186
7	rs1128503	A	G	0.0930
7	rs2032585	C	T	0.0465
7	rs2235030	A	G	0.0697
7	rs10276036	C	T	0.1628
7	rs113125275	G	A	0.1163
7	rs4728702	A	T	0.0930
7	rs28381893	C	T	0.0465
7	rs12704364	T	C	0.4186
7	rs56735241	C	T	0.0930
7	rs6961665	A	C	0.4186
7	rs3789244	G	T	0.1628
7	rs2235028	A	G	0.0465
7	rs2235027	T	G	0.4186
7	rs2235026	T	C	0.1628
7	rs1922240	C	T	0.1628
7	rs139722542	A	G	0.0930
7	rs114106519	C	T	0.0930
7	rs199812223	AT	A	0.0465
7	rs1922241	A	G	0.1628
7	rs57924923	A	G	0.4186
7	rs11772987	G	C	0.1279
7	rs60749469	C	T	0.4186
7	rs10244266	G	T	0.1279
7	rs1882479	G	A	0.1163
7	rs2235023	T	C	0.4186
7	rs28381866	G	A	0.0930
7	rs10274441	G	A	0.1279
7	rs11975994	G	A	0.0930
7	rs28381853	A	G	0.0465
7	rs28381851	G	T	0.0465
7	rs112858219	A	G	0.0697
7	rs28381848	C	T	0.0814
7	rs58525782	C	A	0.0465
7	rs1024409	A	G	0.0697
7	rs201620488_t3	G	T	0.3605
7	rs1989830	A	G	0.3023
7	rs1989829	T	C	0.3023
7	rs73387255	A	G	0.2093
7	rs7809208	A	C	0.3953
7	rs2188526	T	C	0.0465
7	rs3789243	G	A	0.4651
7	rs1858923	G	A	0.0465
7	rs3214119	T	TC	0.0697
7	rs3213619	G	A	0.1395
7	rs28381800	A	T	0.0697
7	rs11395081	AT	A	0.0697
7	rs4148731	A	G	0.0697
7	rs4148730	G	A	0.0697
7	rs28381775	C	T	0.0697
7	rs28381772	T	G	0.0697
7	rs11975403	A	G	0.0581
7	rs10231033	G	A	0.0697
7	rs4148729	G	T	0.0697
7	rs17149840	A	G	0.0697
7	rs75974753	C	T	0.0697
7	rs10280686	T	A	0.0697
7	rs10233247	G	A	0.0814
7	rs10224594	C	T	0.0697
7	rs10275831	T	C	0.0697
7	rs10246878	A	G	0.0581
7	rs200339290	C	CT	0.0930
7	rs10267099	G	A	0.1163
7	rs76348194	A	G	0.0930
7	rs11973812	G	C	0.0697
7	rs11977492	A	T	0.0697
7	rs28483333	T	C	0.0697
7	rs7810499	C	T	0.0697
7	rs28746495	T	C	0.1047
7	rs28746492	G	A	0.0697
7	rs6951067	T	C	0.4884
7	rs142999199	A	AG	0.0697
7	rs78413330	A	G	0.0697
7	rs76190983	A	G	0.0697
7	rs6465117	A	G	0.1977
7	rs10254392	C	T	0.0697
7	rs2106522	G	T	0.1047
7	rs2157930	A	G	0.1163
7	rs58101885	A	G	0.0930
7	rs75910150	T	C	0.0697
7	rs73705296	A	G	0.0930
7	rs12540931	C	T	0.1977
7	rs73705298	A	G	0.0930
7	rs145424538	A	AAAC	0.0697
7	rs77394523	T	C	0.0930
7	rs6957599	A	G	0.0697
7	rs11983274	G	A	0.0697
7	rs7796247	A	G	0.0697
7	rs1015415	T	A	0.2093
7	rs6465118	A	G	0.1977
7	rs10278483	C	T	0.0697
7	rs2188530	G	A	0.0697
7	rs2188529	A	T	0.0697
7	rs201768076	A	AT	0.0930
7	rs17149864	G	A	0.2209
7	rs78854352	T	C	0.0697
7	rs74754276	T	C	0.0697
7	rs11972683	T	C	0.0697
7	rs10232449	A	G	0.0697
7	rs10261685	C	A	0.0697
7	rs10248345	G	T	0.0697
7	rs199704465	T	TTAA	0.0697
7	rs199718071	T	TTA	0.0697
7	rs28572147	T	A	0.0697
7	rs146812933	A	G	0.0697
7	rs10281645	G	A	0.0697
7	rs28774398	A	T	0.1047
7	rs10486997	A	G	0.0697
7	rs12537294	C	T	0.0697
7	rs2188523	A	G	0.0697
7	rs28879532	A	T	0.0697
7	rs10259182	T	C	0.1047
7	rs10275625	T	C	0.0697
7	rs10237438	A	G	0.0697
7	rs78598021	T	C	0.0697
7	rs17149882	A	G	0.0697
7	rs10230766	A	T	0.0697
7	rs10280904	C	T	0.0697
7	rs35378698	CT	C	0.0581
7	rs188284544	T	C	0.0697
7	rs181050063	G	A	0.0697
7	rs28624877	A	T	0.0697
7	rs13437761	T	C	0.0697
7	rs13438341	C	A	0.0697
7	rs10276409	T	G	0.0697
7	rs12532279	G	T	0.1047
7	rs6962399	T	C	0.0697
7	rs13437893	A	G	0.0697
7	rs77994152	G	A	0.0697
7	rs35648408	C	G	0.0581
7	rs147934073	T	TACTTATCCTATG	0.0697
11	rs2712788	T	A	0.2326
11	rs2851076	C	A	0.2209
11	rs2851077	G	A	0.2209
11	rs949071	T	C	0.2209
11	rs80217529	A	G	0.1047
11	rs2851078	G	A	0.2209
11	rs7124613	T	C	0.1047
11	rs2851079	C	T	0.2674
11	rs2851080	C	T	0.2209
11	rs2851081	A	G	0.2209
11	rs2851082	A	T	0.2209
11	rs77953909	G	A	0.1047
11	rs1944615	A	C	0.1163
11	rs4944977	C	T	0.1047
11	rs28806232	T	C	0.0930
11	rs11236332	G	C	0.1628
11	rs2510657	G	A	0.2674
11	rs2513653	A	C	0.2674
11	rs148952117	C	T	0.1047
11	rs2851087	A	G	0.2674
11	rs2513655	C	T	0.2674
11	rs2465291	C	T	0.2674
11	rs2712823	A	G	0.2674
11	rs2851088	C	T	0.2674
11	rs17133681	C	A	0.1047
11	rs2712827	G	A	0.1163
11	rs2712779	A	T	0.2674
11	rs2712780	A	G	0.0581
11	rs4944981	A	G	0.1512
11	rs143837090	A	G	0.0581
11	rs2513656	T	C	0.2442
11	rs2712812	T	C	0.2442
11	rs2851091	A	G	0.3953
11	rs2712803	T	C	0.2442
11	rs2712791	A	G	0.2442
11	rs2712794	A	G	0.4535
11	rs12279394	G	A	0.4186
11	rs144636650	T	C	0.0930
11	rs2712799	A	G	0.1977
11	rs114000664	T	C	0.0930
11	rs79297525	T	C	0.1395
11	rs11236348	A	C	0.407
11	rs11236349	G	T	0.407
11	rs11236351	G	A	0.407
11	rs4100076	C	A	0.1628
11	rs2712807	G	A	0.3023
11	rs2851069	C	T	0.1744
11	rs2712810	T	A	0.3023
11	rs2712819	G	A	0.3023
11	rs2712820	T	C	0.3023
11	rs11236359	A	G	0.4767
11	rs1109407	A	G	0.1279
11	rs1789694	T	C	0.3256
11	rs12422149	A	G	0.0697
11	rs61741839	T	C	0.0465
11	rs1612859	C	T	0.3837
11	rs115881705	C	T	0.0814
11	rs142877598	A	G	0.1047
11	rs3824903	C	A	0.4535
11	rs114169536	T	A	0.1977
11	rs116456559	A	G	0.1047
11	rs112455521	A	G	0.0697
11	rs139480360	A	G	0.0581
11	rs145026521	C	T	0.1047
11	rs149636191	C	G	0.1395
11	rs2306168	C	T	0.5
11	rs139408570	G	T	0.1395
11	rs200583779	G	GT	0.1395
11	rs190362624	A	G	0.1395
11	rs116211275	G	A	0.1395
11	rs114730634	T	C	0.1395
11	rs57141326	A	G	0.0814
11	rs3781727	C	T	0.1744
11	rs41298117	G	C	0.2442
11	rs1801906	C	T	0.4419
11	rs41298121	C	T	0.4419
11	rs17133818	T	C	0.1279
11	rs7951787	G	A	0.4302
11	rs143590827	C	CA	0.1395
11	rs74328774	A	G	0.1395
11	rs57279023	G	A	0.4419
11	rs10793116	G	A	0.0581
11	rs115385770	T	C	0.1395
11	rs148725627	A	G	0.1395
11	rs7924924	C	T	0.2558
11	rs137940642	A	G	0.0581
11	rs77388683	T	A	0.1395
11	rs78028968	C	T	0.1395
11	rs10793117	T	A	0.0465
11	rs147451830	G	A	0.1395
11	rs1676889	T	C	0.0465
11	rs138139803	T	C	0.1395
11	rs138268018	A	C	0.1395
11	rs139792944	G	T	0.1395
11	rs1789679	G	A	0.0465
11	rs76510484	G	C	0.1395
11	rs11825144	G	A	0.0814
11	rs149949680	C	T	0.1395
11	rs145092162	A	G	0.0581
11	rs10899080	A	G	0.0465
11	rs115237508	G	A	0.1395
11	rs115316534	C	T	0.1395
11	rs144143765	T	G	0.1395
11	rs148442723	C	T	0.1395
11	rs142620661	A	C	0.1395
11	rs116234547	C	G	0.1395
11	rs1676891	C	A	0.4767
11	rs116753650	A	G	0.1395
11	rs141666090	T	G	0.1395
11	rs114506297	T	C	0.1395
11	rs76519350	A	G	0.1395
11	rs150487846	A	C	0.1395
11	rs200183315	G	GC	0.1395
11	rs151119066	T	C	0.0465
11	rs145363174	C	G	0.1395
11	rs147485017	G	A	0.1395
11	rs78843443	A	T	0.1395
11	rs148791815	A	T	0.1395
12	rs4149032	C	T	0.1809
12	rs4149056	-	T	0.0
12	rs143218796	A	G	0.0697
12	rs4149069	C	G	0.4884
12	rs76715736	A	G	0.1047
12	rs11045870	A	G	0.0465
12	rs79494188	T	A	0.1047
12	rs11045871	G	A	0.0465
12	rs11045872	G	A	0.0465
12	rs75702089	C	T	0.0465
12	rs10841761	C	G	0.0465
12	rs76373857	C	T	0.1047
12	rs74690297	G	C	0.0465
12	rs11045873	A	T	0.0465
12	rs11045874	C	G	0.0930
12	rs4149079	C	T	0.1512
12	rs12814646	A	C	0.0465
12	rs72655362	A	G	0.1047
12	rs6487214	A	C	0.1512
12	rs7978219	C	T	0.1512
12	rs7965941	G	A	0.1512
12	rs58103058	T	C	0.0465
12	rs7966037	G	A	0.1512
12	rs7966129	T	C	0.1512
12	rs7966448	A	G	0.1512
12	rs7966269	T	C	0.1512
12	rs7966270	C	A	0.0581
12	rs7966581	A	G	0.1512
12	rs7966613	G	A	0.1512
12	rs34882548	AC	A	0.1512
12	rs76928130	T	C	0.0814
12	rs61447271	C	T	0.0930
12	rs7953829	G	T	0.0581
12	rs183892220	T	G	0.0581
12	rs4149082	G	C	0.0930
12	rs58964144	G	T	0.0581
12	rs115108625	A	T	0.0581
12	rs11045877	G	T	0.0930
12	rs12369359	G	T	0.0930
12	rs71444108	GAGT	CCACG	0.2
12	rs78695636	A	C	0.1047
12	rs200875545	C	CAT	0.0814
12	rs10841763	C	T	0.0930
12	rs717959	C	T	0.0930
12	rs115913130	C	T	0.0697
12	rs116180575	A	G	0.0697
12	rs115108635	T	C	0.0697
12	rs3962562	T	G	0.1628
12	rs11045881	C	T	0.1628
12	rs138156422	C	G	0.0814
12	rs11045882	T	C	0.2674
12	rs11045883	A	G	0.0930
12	rs10841764	G	C	0.1628
12	rs10841765	T	C	0.0930
12	rs111359254	C	T	0.0697
12	rs77857357	A	G	0.0697
12	rs78545516	T	C	0.1047
12	rs10841767	G	A	0.0465
12	rs10841768	C	A	0.0465
12	rs11513225	C	T	0.0697
12	rs116472641	G	C	0.1047
12	rs146059821	T	A	0.0697
12	rs201304263	AG	A	0.0465
12	rs12829704	A	G	0.0465
12	rs143654242	G	T	0.0465
12	rs116166170	G	A	0.1047
12	rs11045890	T	C	0.0697
12	rs56164184	C	T	0.0465
12	rs200739289	CT	C	0.1395
12	rs56370646	TA	T	0.1395
12	rs12578392	C	T	0.1395
12	rs57130116	A	G	0.1395
12	rs34111581	C	T	0.0465
12	rs112848945	T	A	0.0465
12	rs11615107	T	C	0.1047
12	rs12371604	C	T	0.0930
12	rs12815795	C	T	0.0465
12	rs34671512	C	A	0.1047
12	rs72655363	T	C	0.1047
12	rs4149087	G	T	0.1395
12	rs4149088	G	A	0.1395
12	rs11045892	G	A	0.0465
12	rs11045893	C	T	0.0465
12	rs12372157	G	T	0.1395
12	rs11045895	C	T	0.0814
12	rs77757956	T	C	0.0697
12	rs12370842	A	G	0.0465
12	rs11045896	C	A	0.0930
12	rs111273303	G	A	0.1047
12	rs115431317	C	A	0.0697
12	rs111436442	G	A	0.1047
12	rs200689244	G	GT	0.0465
12	rs12372593	A	T	0.0465
12	rs74064264	A	C	0.0465
12	rs10841769	A	G	0.3256
12	rs77088330	C	T	0.0697
12	rs116428713	C	A	0.1047
12	rs56397921	G	C	0.0465
12	rs80168561	C	A	0.0697
12	rs7960688	C	G	0.0581
12	rs7960384	G	A	0.0581
12	rs79458194	A	C	0.1047
12	rs78178574	T	G	0.1047
12	rs78083330	C	T	0.1047
12	rs58173663	G	C	0.0465
12	rs56290140	TTAG	T	0.4884
12	rs73248969	T	C	0.0581
12	rs55773124	C	A	0.0581
12	rs11045900	G	A	0.1395
12	rs11045901	C	T	0.1395
12	rs55941487	T	C	0.0465
12	rs114917941	C	A	0.1047
12	rs77256705	G	C	0.0465
12	rs59938822	G	A	0.1047
12	rs201721678	A	ATAACT	0.104
12	rs79036466	G	T	0.1047
12	rs74064273	C	G	0.0465
12	rs73069028	A	G	0.0697
12	rs35325400	C	T	0.1977
12	rs7975166	A	G	0.1047
12	rs35995344	C	T	0.0697
12	rs144440970	T	C	0.1047
12	rs144019604	G	T	0.1047
12	rs77989963	C	T	0.0697
12	rs11045906	G	A	0.2558
12	rs12427132	G	A	0.0697
12	rs145947620	T	G	0.0697
12	rs148455728	A	G	0.0697
12	rs75967989	G	A	0.0581
12	rs10841778	G	T	0.0814
12	rs10841779	C	T	0.1512
12	rs10841780	T	C	0.1512
12	rs71446763	A	G	0.0581
12	rs73250843	A	G	0.1047
12	rs111512821	C	T	0.0697
12	rs10841781	G	A	0.2791
12	rs142851022	G	A	0.0697
12	rs4149009	C	T	0.3256
12	rs78801100	T	C	0.0697
12	rs147803163	A	ATTTGCTTTC	0.1512
12	rs4149008	G	A	0.3837
12	rs4149007	T	C	0.1512
12	rs12317843	C	T	0.4767
12	rs73250847	G	A	0.1395
12	rs4149006	T	G	0.186
12	rs112403792	T	C	0.1744
12	rs11045919	G	T	0.4767
12	rs4140389	G	T	0.4186
12	rs11045920	A	C	0.0581
12	rs11568557	C	G	0.0581
12	rs34636443	ATAAC	A	0.4186
12	rs202105274	ATAAG	T	0.0581
12	rs12230401	C	G	0.1512
12	rs57899519	A	T	0.4186
12	rs16923597	A	G	0.4186
12	rs7980842	T	G	0.2093
12	rs7967354	T	C	0.3837
12	rs10841782	T	C	0.2093
12	rs190500283	A	G	0.0697
12	rs7955581	C	G	0.3721
12	rs150018371	GAGAT	G	0.4186
12	rs2417971	G	A	0.3256
12	rs11045922	C	G	0.3837
12	rs11045923	C	G	0.3605
12	rs16923608	G	T	0.3837
12	rs183479283	A	G	0.0697
12	rs12298817	C	T	0.4186
12	rs143783884	C	A	0.0697
12	rs58923303	T	TGA	0.1395
12	rs12300594	C	T	0.4186
12	rs57503786	T	G	0.2093
12	rs201191507	AAGT	G	0.0581
12	rs4149005	G	T	0.3721
12	rs4149004	C	T	0.0581
12	rs3736081	C	T	0.0581
12	rs12303996	C	T	0.1977
12	rs12297072	A	G	0.0930
12	rs2199688	G	T	0.3837
12	rs11568565	A	G	0.0581
12	rs3764044	C	T	0.1395
12	rs11829484	T	C	0.2093
12	rs112034436	A	G	0.0581
12	rs10431251	T	C	0.4186
12	rs11045926	C	T	0.4186
12	rs11045927	G	T	0.4186
12	rs184309917	C	T	0.0697
12	rs112346601	C	G	0.0581
12	rs113601942	T	C	0.0581
12	rs116763958	C	T	0.0697
12	rs4453284	C	G	0.0581
12	rs61926248	G	C	0.2442
12	rs143498446	A	G	0.0697
12	rs112699138	A	C	0.0581
12	rs7316412	G	C	0.2209
12	rs73250866	C	G	0.1628
12	rs2199687	A	T	0.3837
12	rs112225684	A	G	0.2209
12	rs11045930	T	C	0.407
12	rs77209586	C	T	0.0697
12	rs60594228	C	T	0.1395
12	rs11535999	A	C	0.3256
12	rs116452856	C	T	0.1744
12	rs2417972	C	T	0.407
12	rs113602450	C	T	0.0581
12	rs11833627	C	T	0.314
12	rs112261153	G	T	0.0581
12	rs11533477	A	G	0.0581
12	rs60098288	C	G	0.0814
12	rs184036934	T	C	0.0581
12	rs61552073	A	G	0.1395
12	rs11045941	G	A	0.1279
12	rs79349376	T	A	0.0581
12	rs66777941	C	T	0.3372
12	rs199940208	CGT	C	0.1744
12	rs10841786	A	T	0.3256
12	rs10841787	C	A	0.3256
12	rs74064507	T	A	0.0581
12	rs7305484	A	G	0.1744
12	rs4149003	C	T	0.2558
12	rs74064510	T	G	0.0697
12	rs16923623	G	A	0.0697
12	rs6487215	A	G	0.3372
12	rs7977483	G	A	0.1395
12	rs116040707	G	A	0.1163
12	rs79445965	A	G	0.0581
12	rs7978322	T	G	0.3372
12	rs78735020	A	G	0.2558
12	rs112828357	T	C	0.1744
12	rs10770795	A	G	0.3837
12	rs139780426	A	AAAAT	0.3488
12	rs2900480	G	C	0.3488
12	rs11568574	C	T	0.0581
12	rs11568573	G	A	0.1744
12	rs1000626	G	A	0.0930
12	rs59896213	C	CTT	0.186
12	rs144566343	C	G	0.0697
12	rs4522218	G	T	0.0697
12	rs4148996	G	C	0.2093
12	rs2417973	T	G	0.2093
12	rs7952736	A	G	0.1744
12	rs145614255	A	AACAATTCT	0.2093
12	rs74064512	T	C	0.0581
12	rs139863986	CA	C	0.2093
12	rs11045948	A	G	0.0581
12	rs73235208	T	G	0.2093
12	rs199525542	GTA	G	0.2093
12	rs199877480	C	CCTCT	0.0697
12	rs143814959	ATG	A	0.407
12	rs148416055	A	ATATG	0.1395
12	rs113781670	G	A	0.1744
12	rs11568550	A	G	0.0581
12	rs4148995	C	T	0.0581
12	rs58957989	GT	G	0.1977
12	rs11832851	A	G	0.1977
12	rs11045955	T	A	0.2093
12	rs111334847	T	C	0.1395
12	rs142350951	T	C	0.0697
12	rs11045956	G	A	0.1744
12	rs74064514	C	A	0.1279
12	rs74064516	G	A	0.1279
12	rs114925264	G	A	0.0581
12	rs11045958	G	A	0.186
12	rs11045959	T	C	0.1395
12	rs77676075	A	G	0.1744
12	rs113810619	A	G	0.1744
12	rs112633391	C	A	0.1744
12	rs11838023	C	T	0.2674
12	rs3838822	C	CTG	0.1163
12	rs57472326	A	G	0.3256
12	rs7305911	T	C	0.3256
12	rs151134208	C	T	0.0697
12	rs200037623	T	TTAA	0.0581
12	rs148368950	T	TAATA	0.0581
12	rs80225829	C	T	0.0697
12	rs111254386	G	A	0.1744
12	rs11568567	A	T	0.0697
12	rs4148994	T	C	0.0581
12	rs11045960	A	G	0.1744
12	rs4148993	G	A	0.3256
12	rs11836396	C	T	0.2674
12	rs113449898	C	T	0.1744
12	rs34211424	G	A	0.1744
12	rs112130054	G	C	0.1744
12	rs148678312	A	AAC	0.1744
12	rs12314183	A	G	0.2791
12	rs2417974	C	T	0.0581
12	rs2127117	T	C	0.1163
12	rs2169883	G	A	0.1163
12	rs7304940	C	T	0.1744
12	rs112297403	A	G	0.1977
12	rs199881908	A	AGAG	0.0581
12	rs142521520	CA	C	0.1163
12	rs142749463	T	C	0.0697
12	rs12231484	A	C	0.0581
12	rs2306226	C	T	0.0581
12	rs10841789	C	A	0.1047
12	rs58587133	C	T	0.0814
12	rs61927778	T	C	0.0697
12	rs7137014	C	T	0.1744
12	rs61537911	C	T	0.1512
12	rs77335871	C	T	0.0581
12	rs7974575	A	G	0.1047
12	rs12228765	C	T	0.0581
12	rs12227319	C	G	0.2907
12	rs34249976	C	T	0.1744
12	rs112214775	A	C	0.1977
12	rs59940564	A	T	0.1512
12	rs199577219	GA	G	0.1628
12	rs73237221	T	A	0.0814
12	rs11045966	T	C	0.1163
12	rs58406283	A	G	0.0930
12	rs2219793	G	T	0.2907
12	rs11568570	A	T	0.0581
12	rs2219792	C	G	0.0697
12	rs10505872	A	G	0.0930
12	rs76669231	A	G	0.0581
12	rs11045969	A	C	0.0581
12	rs11833771	G	A	0.1512
12	rs11045971	C	G	0.0581
12	rs10082739	T	C	0.0465
12	rs12298237	T	C	0.1977
12	rs7312628	T	C	0.1512
12	rs61927780	A	G	0.0930
12	rs61927781	C	T	0.0697
12	rs139621920	TA	T	0.0697
12	rs11830993	T	C	0.1395
12	rs4078	G	A	0.0930
12	rs77207093	T	A	0.0930
12	rs113307426	G	A	0.0581
12	rs61927782	A	G	0.0930
12	rs11836945	C	A	0.1512
12	rs61927783	T	C	0.0930
12	rs34696667	A	AT	0.1512
12	rs11832394	T	C	0.1512
12	rs73237235	G	A	0.1512
12	rs114032998	A	G	0.0697
12	rs2199683	A	C	0.1512
12	rs113467449	T	C	0.1512
12	rs143459704	C	CCCCA	0.1512
12	rs11830939	T	G	0.1512
12	rs4148987	A	G	0.4884
12	rs151247881	AAAAT	A	0.1512
12	rs115692334	T	A	0.0581
12	rs61927786	C	T	0.0930
12	rs11832186	G	C	0.1512
12	rs61927787	T	A	0.0930
12	rs61927788	T	C	0.0930
12	rs73237241	C	A	0.1512
12	rs61927789	G	A	0.0930
12	rs112737048	G	C	0.0581
12	rs143086486	G	T	0.0581
12	rs112749642	T	C	0.0581
12	rs113158516	T	G	0.0581
12	rs7968842	G	A	0.3372
12	rs4148986	G	A	0.3372
12	rs4148985	C	T	0.3372
12	rs4148984	A	G	0.3372
12	rs4148983	G	A	0.3372
12	rs4148982	T	C	0.3372
12	rs4148981	C	T	0.3372
12	rs3834939	C	CT	0.3372
12	rs141493226	TATGTAC	T	0.1512
12	rs7980167	T	A	0.0697
12	rs4148978	C	T	0.3372
12	rs4148977	C	T	0.3372
12	rs7980490	T	C	0.0930
12	rs3764042	G	A	0.3372
12	rs12230953	C	T	0.0814
12	rs2857468	A	T	0.3837
12	rs4762818	A	G	0.2674
12	rs11830638	G	A	0.2674
12	rs11838314	C	T	0.2674
12	rs7954757	G	A	0.2674
12	rs1871288	A	T	0.0581
12	rs56348138	T	C	0.2791
12	rs11836617	A	G	0.2674
12	rs7138451	G	A	0.0697
12	rs73069061	A	G	0.2791
12	rs1979405	C	T	0.0581
12	rs56246579	G	A	0.2791
12	rs56806648	T	C	0.2791
12	rs115309986	C	T	0.0814
12	rs75208026	C	T	0.0930
12	rs2061825	T	C	0.0581
12	rs12809856	T	C	0.4767
12	rs12296154	C	T	0.3488
12	rs115685216	G	A	0.0814
12	rs10743413	C	T	0.3721
12	rs11045994	C	T	0.2791
12	rs11045995	C	T	0.0581
12	rs10770800	G	A	0.0465
12	rs73069071	C	T	0.3837
12	rs77397980	C	T	0.0581
12	rs114958964	C	T	0.1512
12	rs114486873	G	A	0.1512
12	rs63378661	G	A	0.1512
12	rs140377659	C	CAATGAGAAAAA	0.1744
12	rs78331403	A	G	0.1163
12	rs5484	T	C	0.1744
12	rs5486	G	A	0.0581
12	rs1056007	T	G	0.1744
12	rs5488	A	T	0.1744
12	rs3213208	G	T	0.1163
12	rs12826421	C	G	0.1744
12	rs12833409	T	C	0.1163
12	rs4762700	C	T	0.4767
12	rs55785938	A	G	0.0581
12	rs12317073	A	G	0.4767
13	rs73551639	A	T	0.2442
13	rs73551640	G	T	0.2442
13	rs73551643	A	G	0.2442
13	rs73551645	A	G	0.2442
13	rs115744968	G	C	0.1628
13	rs9516500	A	G	0.1279
13	rs73551647	A	C	0.2442
13	rs60329452	T	C	0.1628
13	rs9524719	A	G	0.1279
13	rs11841559	G	C	0.2442
13	rs11841597	G	C	0.2442
13	rs10508010	C	A	0.1279
13	rs113234367	A	C	0.1628
13	rs113546805	T	C	0.1628
13	rs112160020	A	T	0.1628
13	rs7996263	T	G	0.2442
13	rs7998670	G	A	0.2442
13	rs113814775	A	G	0.407
13	rs73551665	C	G	0.2442
13	rs111480403	A	G	0.1628
13	rs8002563	A	G	0.407
13	rs7981159	A	G	0.2442
13	rs9590149	G	C	0.4302
13	rs9590150	T	A	0.3372
13	rs6492760	T	C	0.2442
13	rs1925879	C	G	0.4302
13	rs1925878	C	T	0.4302
13	rs1925877	A	G	0.2674
13	rs200517290	A	AT	0.1628
13	rs9302029	C	T	0.4302
13	rs149953938	A	G	0.1628
13	rs9302030	C	T	0.4302
13	rs9302031	C	T	0.4302
13	rs116317258	T	C	0.1628
13	rs1925876	C	T	0.4302
13	rs1925875	G	A	0.2674
13	rs112661898	T	A	0.1628
13	rs16950404	C	T	0.1628
13	rs2148066	G	A	0.4302
13	rs2148065	T	C	0.4302
13	rs1952108	G	A	0.4302
13	rs2148064	G	A	0.4302
13	rs113589545	T	C	0.2442
13	rs141106024	T	C	0.1628
13	rs1925874	A	G	0.2674
13	rs41386448	T	C	0.1628
13	rs1925872	C	A	0.0697
13	rs5805894	AT	A	0.4302
13	rs1925870	T	C	0.4302
13	rs9524731	T	A	0.4302
13	rs9524732	T	C	0.1628
13	rs9302032	A	T	0.186
13	rs72640796	C	G	0.1628
13	rs1925868	A	G	0.3372
13	rs2209537	A	G	0.3372
13	rs34758312	A	G	0.0814
13	rs1925861	A	G	0.1628
13	rs1925860	T	C	0.1628
13	rs1925858	T	C	0.1628
13	rs61965532	A	G	0.1395
13	rs61965533	C	T	0.1395
13	rs9561747	C	T	0.1395
13	rs72642313	T	C	0.1628
13	rs9556446	C	G	0.1395
13	rs9561749	A	G	0.1395
13	rs9556447	A	G	0.1395
13	rs9561750	T	A	0.1395
13	rs9561751	G	T	0.1395
13	rs9561752	A	G	0.1395
13	rs9561753	G	C	0.1395
13	rs34067449	T	A	0.0814
13	rs61965534	G	A	0.1395
13	rs59003216	A	G	0.1395
13	rs71854688	T	TTAAGA	0.139
13	rs9561758	A	G	0.1395
13	rs74107809	A	G	0.0697
13	rs9524741	C	T	0.1395
13	rs9524742	T	C	0.1395
13	rs9524745	A	G	0.2326
13	rs2182264	T	C	0.2326
13	rs11619027	A	G	0.0697
13	rs9590154	G	T	0.2209
13	rs73553674	A	G	0.2093
13	rs9590156	A	G	0.0697
13	rs9556451	T	C	0.0814
13	rs56996395	A	AAT	0.1744
13	rs9561760	A	C	0.407
13	rs111401227	T	C	0.4651
13	rs115036664	A	G	0.0581
13	rs114693747	G	C	0.0697
13	rs9524753	G	C	0.1512
13	rs12863006	A	T	0.186
13	rs60801806	A	T	0.1047
13	rs16950461	C	T	0.1047
13	rs73553699	A	G	0.0930
13	rs7318327	T	C	0.3953
13	rs144138469	A	AAT	0.1047
13	rs111776216	A	C	0.0930
13	rs114211115	C	T	0.1047
13	rs114979997	C	T	0.1047
13	rs74107818	G	A	0.1047
13	rs61043527	T	TAA	0.2442
13	rs9590160	A	C	0.0930
13	rs141851435	G	C	0.1047
13	rs9516518	C	T	0.1163
13	rs61530740	G	A	0.3372
13	rs9590161	G	A	0.1047
13	rs3770	A	G	0.4302
13	rs16950472	G	A	0.1047
13	rs9584273	T	C	0.0697
13	rs1059751	G	A	0.2442
13	rs4148553	T	C	0.2442
13	rs74107819	A	C	0.1047
13	rs115226179	T	C	0.0581
13	rs4148551	T	C	0.3605
13	rs3742106	C	A	0.3372
13	rs4148549	C	T	0.3605
13	rs115345973	T	C	0.0581
13	rs59600142	A	G	0.2791
13	rs61167065	T	C	0.2326
13	rs7331508	G	A	0.186
13	rs112975730	C	G	0.1977
13	rs200689258	AC	A	0.0930
13	rs4773838	C	A	0.4651
13	rs4771904	T	G	0.2674
13	rs9805226	C	T	0.1047
13	rs9302039	T	A	0.4884
13	rs114719300	C	T	0.0581
13	rs75080423	C	A	0.2907
13	rs202160354	CCA	C	0.1047
13	rs4148547	T	C	0.2907
13	rs4148546	G	A	0.3605
13	rs4274307	C	T	0.0697
13	rs4148544	T	C	0.2907
13	rs4148543	G	A	0.314
13	rs61967163	G	A	0.3372
13	rs67308757	G	GA	0.3372
13	rs191458655	A	G	0.0581
13	rs9561765	A	G	0.0814
13	rs116336902	A	G	0.0697
13	rs150571031	C	CAT	0.0697
13	rs7331488	C	T	0.0581
13	rs9302040	A	C	0.2558
13	rs58286099	C	T	0.0814
13	rs112792420	T	A	0.2558
13	rs11568695	T	C	0.1512
13	rs9556455	A	G	0.2558
13	rs9561768	C	T	0.2558
13	rs115100521_t3	G	T	0.0581
13	rs9590168	G	C	0.2558
13	rs9590169	G	A	0.2558
13	rs115850104	G	T	0.0814
13	rs9561769	A	G	0.2558
13	rs200912629	CT	C	0.2558
13	rs139228772	AT	A	0.2558
13	rs113574255	C	G	0.0814
13	rs61967172	T	C	0.2558
13	rs61967173	T	C	0.2558
13	rs114400105	G	T	0.0814
13	rs10219913	C	T	0.2558
13	rs9302043	G	A	0.2558
13	rs61178570	AT	A	0.2558
13	rs7324971	A	G	0.0814
13	rs199553697	CAAAT	C	0.2442
13	rs61967174	T	A	0.2558
13	rs61967175	A	T	0.2674
13	rs7324602	G	A	0.0814
13	rs7324065	T	C	0.0814
13	rs1189449	C	T	0.4535
13	rs113423125	A	T	0.0814
13	rs9302044	G	A	0.1628
13	rs1189445	T	C	0.4186
13	rs1189444	C	T	0.2907
13	rs1614102	C	T	0.3837
13	rs9561773	T	C	0.3256
13	rs9561774	T	C	0.314
13	rs5805899	AT	A	0.1047
13	rs9561776	G	A	0.4535
13	rs3782945	A	G	0.3372
13	rs3782946	C	T	0.4419
13	rs11343244	T	TC	0.1047
13	rs931112	C	A	0.0814
13	rs931111	G	A	0.3372
13	rs922522	G	A	0.3372
13	rs931110	T	C	0.1047
13	rs56695310	A	C	0.0581
13	rs1751033	A	C	0.2326
13	rs1729747	G	C	0.3372
13	rs57270423	C	T	0.3372
13	rs9302045	A	G	0.1047
13	rs11840606	G	A	0.0581
13	rs7997839	G	A	0.1395
13	rs7997756	T	C	0.0814
13	rs201401074	CAG	C	0.1047
13	rs9590171	C	A	0.1047
13	rs150112578	TC	T	0.0814
13	rs1678387	T	C	0.1163
13	rs1678409	T	C	0.1047
13	rs1189457	C	G	0.3256
13	rs1189455	A	G	0.1047
13	rs112582196	A	AG	0.1047
13	rs148638661	A	T	0.0697
13	rs1678392	A	G	0.186
13	rs9524789	A	T	0.2209
13	rs115810488	G	A	0.1047
13	rs1189461	T	C	0.3023
13	rs1189462	C	T	0.1977
13	rs9590172	T	C	0.1047
13	rs1189463	T	C	0.3023
13	rs1751040	C	T	0.3023
13	rs9590173	C	T	0.1047
13	rs9972066	T	C	0.1047
13	rs2274401	C	T	0.4535
13	rs1189464	T	C	0.1279
13	rs1189465	C	T	0.1047
13	rs4148534	C	T	0.4535
13	rs1189466	A	G	0.0814
13	rs1189467	T	A	0.1279
13	rs1189468	A	C	0.1628
13	rs1678339	T	C	0.1279
13	rs1751042	T	G	0.1279
13	rs1751043	A	G	0.0814
13	rs1751045	A	G	0.0814
13	rs1751046	G	A	0.3488
13	rs1617888	T	G	0.3488
13	rs943289	A	G	0.1047
13	rs73557775	A	G	0.0814
13	rs1751051	T	A	0.2791
13	rs2766478	A	T	0.2791
13	rs12867863	A	C	0.0697
13	rs7987858	T	C	0.3721
13	rs1751052	G	A	0.3721
13	rs1189440	G	A	0.1047
13	rs1189439	C	T	0.3721
13	rs1189438	G	A	0.3721
13	rs9590177	T	C	0.1744
13	rs1189436	A	G	0.1047
13	rs1189435	C	T	0.1047
13	rs4148530	T	C	0.0814
13	rs1189434	A	G	0.1047
13	rs34857509	A	G	0.0814
13	rs35904677	A	AT	0.1047
13	rs1189433	C	T	0.1047
13	rs60205363	CA	C	0.0814
13	rs149521078	A	C	0.0697
13	rs145337567	T	C	0.0697
13	rs1617785	G	A	0.2907
13	rs1729760	A	G	0.2907
13	rs4148527	A	G	0.0814
13	rs12584534	T	C	0.0697
13	rs9590184	C	T	0.1744
13	rs145277775	A	G	0.0697
13	rs112327943	A	C	0.0697
13	rs147385814	C	T	0.0697
13	rs4773840	G	A	0.2442
13	rs74105436	G	A	0.2558
13	rs16950650	T	C	0.1395
13	rs1564355	G	A	0.314
13	rs1564354	A	C	0.314
13	rs1564353	C	A	0.314
13	rs143351018	T	C	0.0697
13	rs1751069	A	G	0.0930
13	rs4148515	T	G	0.2907
13	rs4148512	A	G	0.3256
13	rs4148509	T	C	0.3023
13	rs16950656	G	T	0.0465
13	rs57927922	A	G	0.0581
13	rs3782958	C	G	0.3023
13	rs61965885	T	C	0.3023
13	rs114479588	T	G	0.0581
13	rs72643607	A	G	0.0581
13	rs4148506	T	C	0.4884
13	rs1471481	G	A	0.1163
13	rs114964035	A	G	0.0697
13	rs79341676	C	T	0.0814
13	rs1678396	A	G	0.1628
13	rs1750996	A	G	0.4186
13	rs1729764	A	G	0.4186
13	rs1038138	C	T	0.3488
13	rs56261894	C	T	0.1163
13	rs1750999	T	C	0.1512
13	rs7982526	T	G	0.0465
13	rs72643633	T	C	0.0697
13	rs1189458	G	A	0.407
13	rs6492767	T	C	0.2093
13	rs6492768	G	A	0.2093
13	rs1751003	A	G	0.1512
13	rs3864996	C	T	0.2093
13	rs12584917	T	C	0.0581
13	rs4148496	C	T	0.1744
13	rs2478461	T	C	0.1395
13	rs2009772	C	T	0.0581
13	rs1073500	A	C	0.2209
13	rs1751005	T	C	0.1744
13	rs11568662	G	A	0.0930
13	rs55994917	C	A	0.0581
13	rs7330933	G	A	0.407
13	rs61965904	T	C	0.0581
13	rs2793821	T	G	0.2791
13	rs200790129	T	A	0.1395
13	rs4148493	G	T	0.4884
13	rs148748821	CTT	C	0.1395
13	rs118084803	G	A	0.0697
13	rs1887162	T	G	0.1628
13	rs150301651	TCCA	T	0.1047
13	rs4148488	G	C	0.0581
13	rs1564351	G	A	0.0581
13	rs3843689	G	A	0.1628
13	rs2766474	A	G	0.1977
13	rs2148529	G	T	0.0581
13	rs1557070	A	G	0.2442
13	rs4773843	T	C	0.1047
13	rs2487566	G	A	0.4884
13	rs4148482	T	C	0.4535
13	rs1751015	C	T	0.1279
13	rs4148481	A	G	0.4767
13	rs3864997	T	G	0.4767
13	rs1678383	G	T	0.1163
13	rs1678384	A	G	0.2558
13	rs112922776	T	C	0.2209
13	rs9516530	C	T	0.3953
13	rs1678386	C	A	0.3837
13	rs1751021	C	T	0.4767
13	rs1678388	G	A	0.0581
13	rs1751027	G	A	0.2791
13	rs1751029	A	G	0.0581
13	rs1617844	G	A	0.1279
13	rs3818493	G	A	0.4419
13	rs2274405	T	C	0.4419
13	rs2274406	C	T	0.3023
13	rs2274407	A	C	0.2326
13	rs6413442	T	A	0.2209
13	rs3818495	T	A	0.3023
13	rs7331142	C	T	0.4302
13	rs899494	A	G	0.2907
13	rs1678403	C	G	0.3605
13	rs943288	A	T	0.1163
13	rs1678400	A	G	0.1163
13	rs114535776	A	G	0.186
13	rs9524828	C	T	0.4535
13	rs9516537	G	A	0.4535
13	rs9516538	T	C	0.4535
13	rs115962025	T	A	0.0814
13	rs7332663	G	A	0.4535
13	rs7332836	G	A	0.4535
13	rs7333281	G	T	0.2674
13	rs7335147	T	C	0.5
13	rs9516539	A	G	0.5
13	rs9524830	A	T	0.5
13	rs7322825	C	T	0.5
13	rs7139533	A	G	0.5
13	rs35475476	T	TG	0.1163
13	rs9524831	A	C	0.5
13	rs7983336	G	A	0.5
13	rs7987653	T	C	0.5
13	rs9524833	A	G	0.5
13	rs4334136	C	A	0.5
13	rs58721524	A	C	0.0465
13	rs1824913	G	A	0.5
13	rs1824912	A	G	0.5
13	rs1824911	T	A	0.5
13	rs7999175	C	T	0.5
13	rs143604864	C	T	0.186
13	rs9524835	A	G	0.5
13	rs9524836	A	G	0.5
13	rs4773847	T	C	0.5
13	rs201259935	C	CA	0.186
13	rs4148467	T	C	0.5
13	rs34429583	AC	A	0.4535
13	rs34429583_t3	C	A	0.4535
13	rs4771907	C	G	0.5
13	rs4148465	A	G	0.5
13	rs115789275	G	C	0.186
13	rs4148464	G	A	0.5
13	rs4148463	C	G	0.5
13	rs7321744	G	A	0.5
13	rs113500636	CCACTG	C	0.5
13	rs6492769	T	C	0.5
13	rs75557542	G	A	0.5
13	rs114513462	T	C	0.0814
13	rs9524840	A	G	0.5
13	rs115690139	G	A	0.186
13	rs9590203	C	T	0.0697
13	rs4111022	T	C	0.4535
13	rs7321623	A	G	0.5
13	rs11431283	CT	C	0.5
13	rs4771908	C	T	0.5
13	rs67413584	A	AATATT	0.5
13	rs2766476	A	C	0.1163
13	rs9302048	G	C	0.2674
13	rs4773848	A	G	0.3721
13	rs9590204	T	G	0.2674
13	rs9590205	T	A	0.2674
13	rs9302049	C	T	0.2674
13	rs79083026	G	C	0.0697
13	rs114579713	T	C	0.186
13	rs4773849	G	A	0.3721
13	rs1926656	G	A	0.3953
13	rs1926657	T	C	0.3372
13	rs7330519	C	T	0.3837
13	rs7325019	C	T	0.3488
13	rs4612933	T	C	0.3488
13	rs200179424	A	ACT	0.3488
13	rs4371040	T	G	0.3488
13	rs4303338	C	G	0.3488
13	rs9516545	G	A	0.3488
13	rs2892707	T	C	0.3488
13	rs7331366	T	C	0.1279
13	rs4773850	G	T	0.3837
13	rs7330776	A	G	0.0814
13	rs150285358	G	C	0.0697
13	rs114827818	G	A	0.0581
13	rs141735320	A	G	0.0697
13	rs12428470	C	T	0.3488
13	rs116263184	A	T	0.0697
13	rs12854605	G	T	0.3488
13	rs8001451	G	T	0.0697
13	rs58296130	G	A	0.186
13	rs9524845	T	C	0.3488
13	rs4636781	G	A	0.3488
13	rs4505186	G	A	0.3488
13	rs4520712	A	G	0.3488
13	rs4773853	G	A	0.3488
13	rs4773854	G	A	0.3488
13	rs4773855	A	C	0.3488
13	rs6492771	C	T	0.3488
13	rs9590206	T	C	0.0697
13	rs59815481	A	G	0.0581
13	rs7326711	A	G	0.3953
13	rs61972720	G	A	0.1163
13	rs16950823	G	A	0.186
13	rs4148459	C	T	0.1163
13	rs4148456	C	T	0.1163
13	rs4148455	T	C	0.2326
13	rs9590207	A	G	0.0930
13	rs35347010	C	CCTT	0.0930
13	rs114592667	T	C	0.0814
13	rs77037338	A	G	0.1163
13	rs61972725	A	T	0.1163
13	rs2892713	T	C	0.1163
13	rs7337077	T	C	0.0581
13	rs7329532	A	G	0.0581
13	rs7336954	C	T	0.0581
13	rs9590209	A	C	0.0697
13	rs9524859	T	C	0.3372
13	rs9524860	G	T	0.3372
13	rs4479103	C	T	0.2907
13	rs4283094	C	G	0.2907
13	rs56608089	T	C	0.2907
13	rs9524862	G	A	0.2907
13	rs9302051	G	A	0.2907
13	rs9516547	C	T	0.0697
13	rs4148452	A	G	0.2907
13	rs4148451	G	C	0.2907
13	rs4148450	C	T	0.0697
13	rs8003006	G	A	0.2907
13	rs149464244	C	T	0.0465
13	rs6492772	T	C	0.0697
13	rs10508018	A	G	0.1279
13	rs7324464	A	G	0.0697
13	rs7329555	T	G	0.2907
13	rs4771909	T	C	0.2209
13	rs4148445	C	T	0.0814
13	rs4148443	C	T	0.2907
13	rs4148442	T	C	0.2558
13	rs36077567	GA	G	0.2093
13	rs4148441	A	G	0.0697
13	rs4148440	T	C	0.0814
13	rs7335275	C	T	0.1163
13	rs7336202	A	T	0.1163
13	rs8000333	A	G	0.1163
13	rs9524864	T	C	0.0814
13	rs7982955	A	G	0.1163
13	rs4773861	T	C	0.1163
13	rs60115298	C	CT	0.1163
13	rs12584649	C	T	0.3837
13	rs9524866	G	A	0.1163
13	rs60769464	A	C	0.0465
13	rs7320383	G	A	0.1163
13	rs9516550	G	C	0.1512
13	rs73546891	A	G	0.0465
13	rs9556467	C	T	0.1163
13	rs2389227	T	C	0.1163
13	rs2389228	G	A	0.1279
13	rs7319126	A	C	0.2558
13	rs74893509	C	T	0.2209
13	rs76655052	T	G	0.2209
13	rs7320375	G	A	0.2674
13	rs7325256	C	T	0.2674
13	rs7325861	G	T	0.2674
13	rs4148433	C	T	0.2093
13	rs4148432	T	C	0.2674
13	rs4148430	A	T	0.2674
13	rs16950847	T	A	0.2093
13	rs79474727	A	G	0.2093
13	rs9524869	C	G	0.3605
13	rs11842634	C	T	0.2674
13	rs201251479	AC	A	0.1163
13	rs59689275	C	A	0.1163
13	rs11843001	C	T	0.2558
13	rs7338004	A	G	0.0581
13	rs7338429	A	G	0.0581
13	rs7321532	T	C	0.0581
13	rs11843102	G	A	0.0581
13	rs7986087	T	C	0.2558
13	rs7987353	G	A	0.2558
13	rs60223404	T	G	0.0581
13	rs9516552	C	T	0.0814
13	rs79921462	C	T	0.0930
13	rs870004	A	G	0.3721
13	rs7333118	T	G	0.0465
13	rs9590228	C	T	0.3837
13	rs9590229	C	T	0.3837
13	rs7317112	A	G	0.3837
13	rs7322318	T	C	0.3372
13	rs73548830	T	C	0.0465
13	rs150988051	ACATTGC	A	0.1163
13	rs73548833	C	A	0.0465
13	rs7324503	C	A	0.1163
13	rs201187127	AAAAAT	A	0.0465
13	rs112859374	T	C	0.0465
13	rs56891151	G	A	0.0465
13	rs59514866	G	C	0.0465
13	rs4148428	C	T	0.2093
13	rs76888097	T	C	0.0930
13	rs9524873	A	G	0.1395
13	rs9590231	A	G	0.0814
13	rs4773866	T	C	0.1279
13	rs144363729	T	C	0.0581
13	rs9524879	T	C	0.3721
13	rs73548889	C	T	0.0581
13	rs7986283	G	A	0.1512
13	rs7328426	T	C	0.3605
13	rs146331560	A	G	0.0814
13	rs9561830	A	G	0.2558
13	rs147524788	G	A	0.1047
13	rs7330673	G	T	0.2558
13	rs7982930	T	C	0.0814
13	rs871051	T	G	0.407
13	rs8001444	C	T	0.1744
13	rs6650325	A	C	0.1744
13	rs9524896	A	G	0.1628
13	rs61974974	A	G	0.1744
13	rs869951	G	C	0.1744
13	rs2993579	T	C	0.1744
13	rs868853	C	T	0.2674
13	rs1764419	A	G	0.1744
13	rs1764417	T	G	0.186
13	rs9561837	T	C	0.3837
13	rs2992904	C	A	0.314
13	rs2993582	T	G	0.314
13	rs2992905	T	C	0.314
13	rs9302056	T	A	0.314
13	rs9302057	G	A	0.0697
13	rs9524899	A	G	0.3837
13	rs2993583	C	A	0.314
13	rs2992907	T	C	0.2791
13	rs2992908	A	G	0.314
13	rs200012169	A	AAG	0.314
13	rs147591451	A	AG	0.314
13	rs2992909	T	C	0.314
13	rs7332635	A	G	0.3837
13	rs2993584	A	C	0.2791
13	rs2993585	A	G	0.314
13	rs9524901	C	A	0.3837
13	rs2992910	A	T	0.3256
13	rs2993586	C	T	0.2791
13	rs1764410	T	C	0.1628
13	rs7328570	A	G	0.1395
13	rs2993590	C	T	0.1395
13	rs7983929	C	T	0.4767
13	rs8000617	T	C	0.4767
13	rs7984107	A	T	0.4767
13	rs142604921	T	TG	0.0581
13	rs9302061	T	C	0.4767
13	rs9302064	C	A	0.4884
13	rs4773880	T	C	0.0581
13	rs7995750	A	T	0.4767
13	rs4773882	A	G	0.0581
13	rs28843704	T	C	0.0697
13	rs7337939	C	T	0.2326
13	rs66774953	G	T	0.2326
13	rs4773884	G	A	0.4419
13	rs193003058	A	G	0.0814
13	rs9524910	G	A	0.2674
13	rs4773885	G	A	0.4767
13	rs17189568	C	T	0.1163
13	rs9590237	A	G	0.0697
13	rs9524920	A	G	0.3488
13	rs60338761	C	T	0.1047
13	rs2484983	G	A	0.2093
13	rs2993600	G	T	0.407
13	rs73553102	C	T	0.1163
13	rs8001940	A	C	0.2558
13	rs9524925	G	A	0.1744
13	rs1766893	G	A	0.1047
13	rs2993604	C	G	0.1977
13	rs2993605	A	G	0.1977
13	rs2993606	A	G	0.1977
13	rs77146956	C	T	0.1977
13	rs71432038	C	T	0.1977
13	rs66489010	G	A	0.1977
13	rs28671610	C	T	0.1977
13	rs16950985	T	G	0.1977
13	rs2389257	A	C	0.1977
13	rs2389256	G	A	0.1977
13	rs1764430	G	C	0.1977
13	rs1766912	G	A	0.186
13	rs1766911	G	A	0.186
13	rs2389254	G	A	0.1977
13	rs61478273	CAG	C	0.1977
13	rs2485972	A	G	0.1977
13	rs12875123	A	T	0.1977
13	rs2993554	G	A	0.1977
13	rs2389253	C	T	0.1977
13	rs116182952	G	T	0.1628
13	rs2992886	A	G	0.2093
13	rs2993555	T	C	0.2093
13	rs2992887	C	A	0.2093
13	rs114675293	T	A	0.1628
13	rs2992888	G	A	0.1977
13	rs1620972	A	G	0.1977
13	rs149181983	AT	A	0.1628
13	rs11619347	A	G	0.1628
13	rs10508015	C	T	0.2209
13	rs12584768	C	T	0.1977
13	rs9524936	G	C	0.1977
13	rs9524937	G	T	0.1977
13	rs9516568	G	A	0.4535
13	rs9516572	T	C	0.1977
19	rs200039365	G	GC	0.1047
19	rs60533951	A	G	0.1047
19	rs7937	T	C	0.2326
19	rs76268776	T	C	0.0581
19	rs2545761	T	C	0.1279
19	rs2545763	A	G	0.0930
19	rs79692712	G	C	0.0581
19	rs11666504	T	C	0.0930
19	rs16974537	A	G	0.0465
19	rs10405596	T	C	0.2558
19	rs2545769	G	A	0.4767
19	rs2545771	A	G	0.0930
19	rs2545772	A	G	0.0814
19	rs11879672	T	C	0.4767
19	rs28483485	T	C	0.4767
19	rs112673025	A	G	0.0465
19	rs79366653	G	A	0.0581
19	rs11878604	C	T	0.2907
19	rs55978439	A	T	0.0465
19	rs2258314	T	C	0.0697
19	rs10853742	G	C	0.1977
19	rs12327581	T	C	0.4419
19	rs11667314	T	C	0.1977
19	rs7251418	A	G	0.1047
19	rs76112798	T	C	0.0697
19	rs7248240	G	C	0.1163
19	rs56164728	C	T	0.0697
19	rs28399462	A	G	0.0697
19	rs4803380	T	C	0.0697
19	rs28399454	T	C	0.0697
19	rs144437384	A	G	0.0697
19	rs72549444	A	G	0.0697
19	rs56113850	C	T	0.2791
19	rs28399433	C	A	0.0697
19	rs61663607	C	T	0.1512
19	rs111822043	A	T	0.0465
19	rs8102683	T	C	0.2209
19	rs8105704	T	C	0.2093
19	rs12610432	T	C	0.2093
19	rs186830274	C	T	0.0581
19	rs111867898	C	T	0.0465
19	rs4570983	T	C	0.1279
19	rs75152309	A	T	0.0697
19	rs74493998	T	C	0.0697
19	rs28575771	A	G	0.2209
19	rs2261144	G	A	0.1512
19	rs12975382	T	G	0.4535
19	rs73032311	C	T	0.0697
19	rs73032316	C	G	0.0697
19	rs3815706	G	T	0.0697
19	rs56081734	A	C	0.4535
19	rs66882672	G	A	0.0814
19	rs67808403	G	A	0.0814
19	rs149560129	G	A	0.0465
19	rs4803393	C	T	0.1163
19	rs79809963	C	A	0.0814
19	rs76734307	C	T	0.0814
19	rs10853743	T	C	0.0697
19	rs3875155	C	T	0.0814
19	rs4105141	A	T	0.2326
19	rs5007415	A	C	0.2326
19	rs10411264	T	C	0.2326
19	rs115564457	T	C	0.1279
19	rs28472879	A	G	0.2326
19	rs28463685	A	G	0.1395
19	rs10406188	G	A	0.2326
19	rs8103288	G	C	0.2326
19	rs8103444	C	A	0.0697
19	rs10414481	T	C	0.2326
19	rs78374326	G	T	0.2326
19	rs3865457	T	C	0.2326
19	rs12611183	A	T	0.0814
19	rs199970591	T	TATCA	0.0814
19	rs76297159	G	A	0.0814
19	rs12609982	G	A	0.0814
19	rs12608615	C	T	0.0814
19	rs34127861	TA	T	0.0814
19	rs73931385	C	A	0.0697
19	rs142357867	T	G	0.0348
19	rs73931386	A	G	0.0697
19	rs9630870	A	G	0.0581
19	rs6508953	A	G	0.0814
19	rs150311873	T	C	0.0465
19	rs7252852	T	C	0.3372
19	rs66657317	G	GT	0.0814
19	rs3852871	C	A	0.0697
19	rs201010762	G	GA	0.0697
19	rs17726493	T	C	0.0814
19	rs55790533	A	G	0.0814
19	rs113921300	C	T	0.0465
19	rs34724660	A	G	0.0697
19	rs3892666	C	G	0.1628
19	rs78367667	G	A	0.0697
19	rs73034462	A	G	0.3023
19	rs73034465	G	A	0.3023
19	rs7252501	T	C	0.1628
19	rs4358050	A	G	0.1628
19	rs4359558	A	G	0.3023
19	rs112531545	G	C	0.1628
19	rs4803404	A	C	0.1628
19	rs4468739	C	T	0.3023
19	rs4001944	A	C	0.3023
19	rs55779134	GT	G	0.3023
19	rs4001941	G	A	0.4767
19	rs34418474	T	G	0.0697
19	rs12459860	C	T	0.0697
19	rs57274441	G	T	0.3023
19	rs4609955	C	T	0.3023
19	rs12150973	C	T	0.3023
19	rs7255901	C	T	0.3023
19	rs12459233	G	C	0.0697
19	rs35781447	A	G	0.0697
19	rs12985721	G	A	0.0697
19	rs28687008	T	C	0.1512
19	rs145709497	C	T	0.0930
19	rs34151237	G	T	0.4186
19	rs34013487	G	C	0.2093
19	rs8107329	T	C	0.3023
19	rs74723889	T	C	0.0930
19	rs8100958	C	T	0.314
19	rs4124633	C	T	0.186
19	rs7245500	C	A	0.3837
19	rs11667592	C	T	0.0814
19	rs8109818	G	A	0.3837
19	rs73557157	A	C	0.2442
19	rs61586981	G	A	0.2442
19	rs60618302	T	A	0.2558
19	rs111588961	T	C	0.2442
19	rs73933714	T	G	0.4651
19	rs35950631	C	A	0.3953
19	rs16974790	A	G	0.3953
19	rs73933721	A	G	0.3953
19	rs16974794	G	A	0.3953
19	rs73559241	G	A	0.3953
19	rs8100458	C	T	0.186
19	rs59243457	T	C	0.3256
19	rs1872125	C	T	0.3256
19	rs8101756	C	T	0.3256
19	rs8104022	A	C	0.3256
19	rs10409285	T	C	0.3256
19	rs62109048	C	T	0.3256
19	rs7250601	C	A	0.3256
19	rs2873264	T	C	0.1628
19	rs11672911	A	G	0.1628
19	rs2279341	C	G	0.0814
19	rs4803418	G	C	0.1163
19	rs12985017	C	T	0.0814
19	rs12985269	C	T	0.0814
19	rs4803419	T	C	0.0930
19	rs3745274	T	G	0.2907
19	rs2279345	T	C	0.1512
19	rs6508965	T	C	0.1512
19	rs6508966	G	C	0.1512
19	rs28399499	C	T	0.1163
19	rs8192719	T	C	0.2907
19	rs36118214	A	G	0.3256
19	rs11671243	A	C	0.1512
19	rs7260329	A	G	0.1047
19	rs7246465	T	C	0.1977
19	rs707265	A	G	0.1512
19	rs200843564_t3	C	A	0.3023
19	rs1042389	C	T	0.3023
19	rs56777936	G	C	0.0814
19	rs36002231	C	T	0.2093
19	rs34299754	G	T	0.2093
19	rs1552220	A	G	0.4302
19	rs1552221	C	T	0.4302
19	rs1552222	A	T	0.314
19	rs2113103	A	G	0.1047
19	rs60554840	T	G	0.1047
19	rs55869705	A	G	0.2326
19	rs10401226	G	A	0.2907
19	rs11666982	T	G	0.1512
19	rs112677106	C	T	0.2326
19	rs11670865	T	G	0.2907
19	rs7249735	C	A	0.2907
19	rs3745275	A	G	0.2907
19	rs7254767	G	C	0.3023
19	rs34855348	A	G	0.1047
19	rs73561518	G	A	0.1163
19	rs17799912	T	C	0.1047
19	rs113129391	T	G	0.1163
19	rs16974869	C	T	0.2326
19	rs73933730	T	G	0.2326
19	rs149490306	A	C	0.0697
19	rs16974893	G	A	0.2326
19	rs7250597	T	C	0.2326
19	rs12982859	A	G	0.1047
19	rs73561542	T	G	0.0581
19	rs11672352	G	A	0.1628
19	rs10407196	G	C	0.0814
19	rs13344213	A	C	0.0814
19	rs113161508	A	G	0.0581
19	rs201853514	TC	T	0.2558
19	rs28502605	C	T	0.2558
19	rs7255149	C	A	0.4535
19	rs12459147	G	A	0.4651
19	rs34150638	A	G	0.4535
19	rs202055073	AT	A	0.0697
19	rs11672085	T	C	0.2791
